# MBD4 loss results in global reactivation of promoters and retroelements with low methylated CpG density

**DOI:** 10.1186/s13046-023-02882-z

**Published:** 2023-11-14

**Authors:** Christophe Papin, Abdulkhaleg Ibrahim, Jamal S. M. Sabir, Stéphanie Le Gras, Isabelle Stoll, Raed S. Albiheyri, Ali T. Zari, Ahmed Bahieldin, Alfonso Bellacosa, Christian Bronner, Ali Hamiche

**Affiliations:** 1 Institut de Génétique Et Biologie Moléculaire Et Cellulaire (IGBMC), UdS, CNRS, INSERM, Equipe Labélisée Ligue Contre Le Cancer, 1 Rue Laurent Fries, B.P. 10142, Illkirch, 67404 Cedex, France; 2 National Research Centre for Tropical and Transboundary Diseases (NRCTTD), Alzentan, 99316 Libya; 3 Centre of Excellence in Bionanoscience, King Abdulaziz University, Jeddah, Saudi Arabia; 4 Department of Biological Sciences, Faculty of Science, King Abdulaziz University, Jeddah, Saudi Arabia; 5 Cancer Biology Program, Cancer Epigenetics Program, Fox Chase Cancer Center, Philadelphia, PA 19111 USA

**Keywords:** MBD4, MLH1, Mismatch repair, Cancer, DNA methylation

## Abstract

**Background:**

Inherited defects in the base-excision repair gene MBD4 predispose individuals to adenomatous polyposis and colorectal cancer, which is characterized by an accumulation of C > T transitions resulting from spontaneous deamination of 5’-methylcytosine.

**Methods:**

Here, we have investigated the potential role of MBD4 in regulating DNA methylation levels using genome-wide transcriptome and methylome analyses. Additionally, we have elucidated its function through a series of in vitro experiments.

**Results:**

Here we show that the protein MBD4 is required for DNA methylation maintenance and G/T mismatch repair. Transcriptome and methylome analyses reveal a genome-wide hypomethylation of promoters, gene bodies and repetitive elements in the absence of MBD4 in vivo. Methylation mark loss is accompanied by a broad transcriptional derepression phenotype affecting promoters and retroelements with low methylated CpG density. MBD4 in vivo forms a complex with the mismatch repair proteins (MMR), which exhibits high bi-functional glycosylase/AP-lyase endonuclease specific activity towards methylated DNA substrates containing a G/T mismatch. Experiments using recombinant proteins reveal that the association of MBD4 with the MMR protein MLH1 is required for this activity.

**Conclusions:**

Our data identify MBD4 as an enzyme specifically designed to repair deaminated 5-methylcytosines and underscores its critical role in safeguarding against methylation damage. Furthermore, it illustrates how MBD4 functions in normal and pathological conditions.

**Supplementary Information:**

The online version contains supplementary material available at 10.1186/s13046-023-02882-z.

Inherited defects in the base-excision repair gene MBD4 predispose individuals to adenomatous polyposis and colorectal cancer Which is characterized by an accumulation of C > T transitions resulting from spontaneous deamination of 5’-methylcytosine. despite its significance This DNA repair pathway is still poorly understood. Here we show that the protein MBD4 is required for DNA methylation maintenance and G/T mismatch repair. Transcriptome and methylome analyses reveal a genome-wide hypomethylation of promoters Gene bodies and repetitive elements in the absence of MBD4 in vivo. Methylation mark loss is accompanied by a broad transcriptional derepression phenotype affecting promoters and retroelements with low methylated CpG density. MBD4 in vivo forms a complex with the mismatch repair proteins (MMR), Which exhibits high bi-functional glycosylase/AP-lyase endonuclease specific activity towards methylated DNA substrates containing a G/T mismatch. Experiments using recombinant proteins reveal that the association of MBD4 with the MMR protein MLH1 is required for this activity. the described data identify MBD4 as an enzyme specifically designed to repair deaminated 5-methylcytosines and illustrates how MBD4 functions in normal and pathological conditions

## Introduction

DNA methylation is an essential epigenetic modification in vertebrates. Implicated in both gene expression and genome stability, it represses promoter activity and prevents the relocation of transposable elements. DNA methylation occurs predominantly in CpG dinucleotides to produce 5-methylcytosines (5mC), which can also be further oxidized by the Ten eleven translocation (TET1, 2 and 3) enzymes [1–4] to 5-hydroxymethylcytosine (5hmC), 5-formylcytosine (5fC) and 5-carboxylcytosine (5caC). 5mC are highly sensitive to spontaneous deamination which leads to the formation of thymine and thus to point mutations [5, 6]. The genome of vertebrates is consequently largely CpG-deficient. The globally methylated and CpG-poor genomic landscape is, however, punctuated by CpG-rich regions referred to as CpG islands (CGIs) [7, 8]. Most of CGIs colocalize with promoters [9] and are protected from methylation, creating transcriptionally permissive chromatin [10]. However, there are well-known examples of CGIs that become methylated during development, in a tissue-specific manner, leading to stable silencing of the associated promoters [11–13].

The inhibitory effect of DNA methylation on transcription is mediated by two main mechanisms. First, the methyl group can directly affect both the recognition and the binding of transcription factors. The second mechanism is indirect and involves the recruitment of transcription repressive complexes to methylated DNA through methyl-CpG binding proteins (MBPs) [14, 15].

Three different families of MBPs are identified: the MBD (Methyl Binding Domain) family, the zinc finger family and the SRA family [16, 17]. The structure of several members (alone or in complex with methylated DNA) of these families were solved by either solution NMR spectroscopy or by X-ray crystallography [18–24]. The available data show that the MBPs recognize and bind to methylated DNA in a very specific manner. Intriguingly, the discrimination between methylated and non-methylated DNA is achieved via distinct MBPs protein folds [25].

The MBD family is composed of seven members, and four of them (MeCP2, MBD1, MBD2 and MBD4) are shown to preferentially bind to methylated DNA through their conserved MBD [26]. Among both the MBD family and the other two families of MBPs, MBD4 is the only protein which exhibits enzymatic activity. Indeed, MBD4, in addition to its methyl-binding domain, has a glycosylase domain and possesses thymine and uracil glycosylase activity [27–29]. Note that MBD4 was cloned in the past by a two-hybrid approach using MLH1, a mismatch repair (MMR) protein, as a “bait” [27]. The DNA mismatch repair system depends, in addition to MLH1, on several other factors, including the proteins MSH2, PMS2 and MSH6 [30]. Reduced levels of MLH1, PMS2, MSH2 and MSH6 were detected in *Mbd4*-deficient cells, suggesting that MBD4 might be involved in both the integrity and stability of the MMR complex [31].

The available data suggest that MBD4 is implicated in Base Excision Repair (BER) of G/T mismatches resulting from deamination of 5mC at CpG dinucleotide. This should avoid mutations and maintain genome stability. In agreement with this, *Mbd4*^−/−^ mice exhibit a marked increase in C to T mutations at CpG sites and higher occurrence of these mutations has been demonstrated in colon tumors in crosses of *Mbd4*^−/−^ mice with *Apc*^Min^ mice [32, 33]. Germline MBD4 deficiency causes uveal melanoma and multi-tumor predisposition syndrome [34–36]. In addition, between 26 and 43% of human gastric, colorectal, endometrial and pancreatic tumors exhibiting microsatellite instability have also mutations in MBD4 [37–41].

Here, we have studied the potential role of MBD4 in the regulation of DNA methylation level in vivo and have deciphered its function in a series of in vitro experiments. Genome-wide transcriptome and methylome analyses reveal that the absence of MBD4 correlates with both hypomethylation and derepression of a large number of genes as well as retro-elements with low methylated-CpG density. We show that MBD4 is associated in vivo with several proteins, including the MMR proteins MLH1 and PMS2. The MBD4 complex exhibits both bifunctional glycosylase/AP (apurinic or apyrimidinic site)—3'-phosphomonoester lyase activity and marked preference for methylated DNA. Experiments using recombinant proteins reveal that the association of MBD4 with the MMR protein MLH1 is required for this activity. Our data identify MBD4 as a key factor designed to repair the product of 5mC deamination (G/T mismatches) in the vicinity of methylated CpG, to maintain methylated chromatin in a repressive state.

## Materials and methods

### Isolation of MEFs

Primary WT or KO MEFs for *Mbd4* were isolated from *Mbd4*^+/+^ and *Mbd4*^−/−^ mice respectively, as previously described [31].

### RNA-seq

After isolation of total cellular RNA from subconfluent MEFs (two independent samples were purified from both WT or KO MEFs for *Mbd4*), libraries of template molecules suitable for strand specific high throughput DNA sequencing were created using “TruSeq Stranded Total RNA with Ribo-Zero Gold Prep Kit” (# RS-122–2301, Illumina). Briefly, starting with 300 ng of total RNA, the cytoplasmic and mitochondrial ribosomal RNA (rRNA) were removed using biotinylated, target-specific oligos combined with Ribo-Zero rRNA removal beads. Following purification, the RNA was fragmented into small pieces using divalent cations under elevated temperature. The cleaved RNA fragments were copied into first strand cDNA using reverse transcriptase and random primers, followed by second strand cDNA synthesis using DNA Polymerase I and RNase H. The double stranded cDNA fragments were blunted using T4 DNA polymerase, Klenow DNA polymerase and T4 PNK. A single ‘A’ nucleotide was added to the 3’ ends of the blunt DNA fragments using a Klenow fragment (3' to 5'exo minus) enzyme. The cDNA fragments were ligated to double stranded adapters using T4 DNA Ligase. The ligated products were enriched by PCR amplification (30 s at 98 °C; [10 s at 98 °C, 30 s at 60 °C, 30 s at 72 °C] × 12 cycles; 5 min at 72 °C). Then surplus PCR primers were removed by purification using AMPure XP beads (Agencourt Biosciences Corporation). Final cDNA libraries were checked for quality and quantified using 2100 Bioanalyzer (Agilent). The libraries were loaded in the flow cell at a concentration of 7 pM, and clusters were generated in the Cbot and sequenced in the Illumina Hiseq 2500 as single-end 50 base reads following Illumina's instructions. Image analysis and base calling were performed using RTA 1.17.20 and CASAVA 1.8.2. Reads were mapped onto the mm9 assembly of the mouse genome by using Tophat [42] and the bowtie aligner [43]. Quantification of gene expression was performed using HTSeq (http://www-huber.embl.de/users/anders/HTSeq) and gene annotations from Ensembl release 67. Read counts have been normalized across WT and KO libraries with the statistical method proposed by Anders and Huber [44] and implemented in the DESeq Bioconductor library. Resulting *p*-values were adjusted for multiple testing by using the Benjamini and Hochberg method [45].

### Clonal bisulfite sequencing and RRBS

Genomic DNA was isolated from subconfluent primary MEFs as previsously described [46]. Digested DNA (500 ng) was converted with EZ DNA Methylation-Gold Kit (Zymo Research Corporation). Primer design was accomplished using Methprimer. Bisulfite sequencing primers (5′-TTTTTTTATGAATAAGTAATTTAATAATAT-3′ and 5′-AATTTCCTAAAATCCCAAATCTCTC-3′ for *Zic5*, 5’- GTTGGAGGTGATTAGGGTTTAAAA-3’ and 5’- TCTAATCAAAAAAACTCCCTAAACC for *Bzrap1*) were used to amplify the corresponding promoters. PCR included an initial incubation at 95 °C for 10 min, followed by 40 cycles of 95 °C for 30 s, 52 °C for 30 s, and 72 °C for 60 s, followed by one cycle of 72 °C for 10 min. The PCR products were cloned into the pCR2.1-TOPO vector using the TOPO TA cloning kit (Invitrogen) for sequencing. A total of 10 clones from each sample were sequenced at the GATC Biotech company, and the methylation status for each CpG site was determined by assessing the presence of T (unmethylated) versus C (methylated) at each CpG site.

For RRBS, bisulfite-converted genomic DNA libraries were prepared according to the previously described methods [47]. Briefly, genomic DNA was digested with MspI (New England Biolabs), followed by end-repair and addition of 3′ A overhangs. Methylated adaptors (Illumina) with a 3′ T overhang were ligated to the A tailed DNA fragments. For reduced representation, 40 to 220 bp (pre-adaptor-ligation size) fragments were excised from 2% TAE agarose gels and bisulfite-converted with EZ DNA methylation Gold kit (Zymo Research). Bisulfite converted libraries were amplified by PCR and sequenced on an Illumina HiSeq 2000 sequencer with a single-ended, 49 bp run (Beijing Genomics Institute). FASTQ sequence files containing sequenced reads were obtained for both samples (WT or KO MEFs for *Mbd4*).

### RRBS data processing

After removal of the adaptor sequences, the 49 bp reads from each sample were aligned to the reference genome (mm9) as well as the size-selected MspI fragments generated by our *in-silico* simulation. Because of the strand specificity of DNA methylation, two rounds of alignments were carried out, i.e. the bisulfite converted reads were aligned to the genome sequences termed the “T genome” with each cytosine converted to thymine, and simultaneously the reads were also aligned to the genome sequences termed the “A genome” with each guanine converted to adenosine. The alignments were carried out with BAMAP aligner allowing up to two mismatches for successful mapping. Summary of the data quantity after each step of filtration is shown in Supplementary Fig. 1A.

### Bioinformatic analyses

The mouse CGI database was retrieved from the UCSC Genome Bioinformatics site using Table Browser program (genome: mouse; assembly: NCBI37/mm9; group: Expression and Regulation; track CpG islands). The mouse promoter database was produced using promoter sequences consisting of the—2 kb to 0 genomic interval relative putative TSSs, using the start of the RefSeq. The percentage of CG methylation was determined by obtaining the number of methylated CGs divided by the total number of CpG dinucleotides per region. The calculation of CG methylation percentages was limited to CGIs and promoters with a minimum 80% coverage of CpG sites. For each CpG site, the methylation level of 5mCG was calculated as the ratio of methylated reads to the number of total reads covered that CpG. We only considered regions with an average coverage of at least 5 reads per methylated CpG to calculate the average methylation level of 5mCpG in CGIs and promoters.

### 5mC/5hmC DNA immunoprecipitation assays

DNA immunoprecipitation assays were done as previously described [48]. Briefly, 10 µg of DNA was used as input, and 2 µl of 5mC antibody (Active Motif, 39,649) or 4 µl of 5hmC antibody (Active Motif, 39,791) was used to immunoprecipitate modified DNA. DNA and antibodies were incubated at 4 °C overnight in a final volume of 500 µl of DIP buffer (10 mM sodium phosphate pH 7.0, 140 mM NaCl, 0.05% Triton X-100). The bound material was recovered after incubation with 30 µl of blocked protein G Dynabeads (beads washed three times with 1 ml of DIP buffer and incubated for 4 h minimum with BSA 1 mg ml^−1^ and yeast tRNA 0.5 mg ml^−1^). The beads were washed three times with 1 ml of DIP buffer, then treated overnight with RNase at 65 °C in presence of 300 mM NaCl and then treated 4 h with proteinase K at 55 °C. Immunoprecipitated DNA was purified by phenol–chloroform extraction followed by ethanol precipitation. Four independent DNA immunoprecipitations were pooled for each condition before sequencing analysis. Sequencing was performed on an Illumina Hiseq 2500 as single-end 50 base reads following Illumina’s instructions. Image analysis and base calling were performed using RTA 1.17.20 and CASAVA 1.8.2. Reads were mapped to the mouse genome (mm9) using bowtie [43] with the following arguments “-m 1 –strata –best -y -S -l 40 -p 2”. We extract the tag density in a 2 kb window surrounding the gene bodies running seqMINER (http://bips.u-strasbg.fr/seqminer/), using datasets normalized to the total number of uniquely mapped reads. Tag densities were calculated in 100 bp sliding windows spanning ± 2 kb of gene bodies. For average gene profiles, genes were divided in 40 bins of length relative to the gene length, and the adjacent 2 kb sequences in 10 bins.

### Repeat analysis

Repeat analyses of RNA-seq and DIP-seq datasets were performed as follows. Reads were aligned to repetitive elements in two passes. In the first pass, reads were aligned to the non-masked mouse reference genome (NCBI37/mm9) using BWA [49] v0.6.2. Positions of the reads uniquely mapped to the mouse genome were cross-compared with the positions of the repeats extracted from UCSC (RMSK table in UCSC database for mouse genome mm9) and reads overlapping a repeat sequence were annotated with the repeat family. In the second pass, reads not mapped or multi-mapped to the mouse genome in the previous pass were aligned to RepBase [50] v18.07 repeat sequences for rodent. Reads mapped to a unique repeat family were annotated with their corresponding family name. Finally, we summed up the read counts per repeat family of the two annotation steps. Data were normalized based upon library size. Difference of repeat read counts between samples was expressed as the log_2_-ratio KO/WT. The statistical significance of the difference between samples was assessed using the Bioconductor package DESeq. Processed datasets presented in this paper were restricted to retrotransposon families (SINEs, LINEs and LTRs) with more than 100 mapped reads per DIP/RNA sample to avoid over- or underestimating fold enrichments due to low sequence representation.

### cDNA clones and construction of mutants

Full-length human cDNA clones of MBD4 (IMAGE 3534047), MLH1 (IMAGE 3451538) and PMS2 (IMAGE 7939766) were purchased from Source BioScience. The coding sequence of MBD4 was mutated using megaprimer PCR to produce MBD4 R97G and MBD4 D554A mutant proteins.

### Cell line generation and complex purification

MBD4 full-length cDNA was subcloned into the XhoI-NotI sites of the pOZ-N retroviral vector to produce MBD4 protein fused with N-terminal Flag- and HA-epitope tags (e-MBD4). e-MBD4 was stably expressed in HeLa cells or in MEFs by retroviral transduction [51]. e-MBD4 nuclear complex (e-MBD4.com) was purified from these cells by double immunoaffinity purification as previously described [52]. MBD4 concentration in e-MBD4.com was estimated by polyacrylamide gels silver-staining using His-tagged MBD4 protein as a standard. Identification of proteins was carried out by Taplin Biological Mass Spectrometry Facility (Harvard Medical School, Boston, MA).

For glycerol density gradient, samples were loaded onto a 4.5 mL glycerol gradient (15%-35%) and spun at 45,000 rpm in a Beckman SW60 rotor for 16 h. Fractions were collected from the bottom of the tube.

### Antibodies

Antibodies employed were as follows: monoclonal antibodies anti-Flag M2-Peroxidase (Sigma), anti-TIP49B (612,482, BD Transduction), anti-MLH1 (NA28, Calbiochem), anti-PMS2 (556,415, BD PharMingen), anti-TIP49A (ab51500, Abcam); polyclonal antibody anti-MBD4 (A-1009, Epigentek).

### Purification of MBD4 recombinant protein

The His-tagged protein was cloned in pET28b vector and expressed in the BL21-CodonPlus-RIL-pLysS (Stratagene) strain. An 800 mL culture was grown in LB medium at 37 °C until D_600_ of 0.5 was reached before induction with 100 µM IPTG for 2 h at 25 °C. Cells were lysed in 20 mL of buffer containing 10 mM Tris–HCl pH 7.65, 500 mM NaCl, 10% glycerol, 0.01% NP40, 10 mM Imidazole, 0.2 mM PMSF and protease inhibitor cocktail tablets (Roche) on ice in the presence of lysozyme at 1 mg/mL and sonicated on ice for 3 × 1 min. His-tagged proteins were immunoprecipitated from clarified supernatant with Ni–NTA-agarose (Qiagen), washed with 50 mM Imidazole and eluted with 300 mM Imidazole using a buffer containing 10 mM Tris–HCl pH 7.65, 150 mM NaCl, 10% glycerol, 0.01% NP40. The eluate fraction was diluted two times with sodium phosphate buffer (50 mM sodium phosphate pH 7.0, 1 mM DTT, 1 mM EDTA), incubated with SP sepharose fast flow beads (GE Healthcare), extensively washed with sodium phosphate buffer containing 300 mM NaCl and eluated with sodium phosphate buffer containing 500 mM NaCl. The eluate fraction was desalted with PD-10 Sephadex G-25 columns (GE Healthcare) equilibrated with TGEN buffer containing 150 mM NaCl. His-tagged MBD4 purified proteins were quantified using the Bradford assay with BSA as a standard.

### Purification of MBD4/MLH1 and MBD4/MLH1/PMS2 complexes

Flag-tagged MBD4, His-tagged MLH1 and HA-tagged PMS2 proteins were cloned in pFastBac vector (Invitrogen). The cloned vectors were transformed into bacterial DH10Bac competent cells for making recombinant bacmid. The recombinant bacmid was then extracted and transfected into Sf9 cells by Cellfectin II Reagent (Invitrogen). After viral amplification, SF9 cells were infected (10^6^ cells per mL) with baculoviruses expressing either Flag-MBD4 alone or in combination with His-MLH1 and/or HA-PMS2 for 2 days at 27 °C. Cells were harvested and resuspended in 25 mL lysis buffer containing 10 mM Tris–HCl pH 7.65, 500 mM NaCl, 10% glycerol, 0.01% NP40, 20 mM Imidazole, 0.2 mM PMSF and protease inhibitor cocktail tablets (Roche). The lysate was dounced 30 times, sonicated, and centrifuged for 10 min at 12,000 rpm. The clarified supernatant was incubated at 4 °C with anti-FLAG M2 affinity agarose resine (SIGMA), washed 3 times with lysis buffer and 3 times with wash buffer containing 10 mM Tris–HCl pH 7.65, 500 mM NaCl, 10% glycerol, 0.01% NP40 and 40 mM Imidazole. The immunoprecipated proteins were eluted with Flag peptide (0.5 mg/mL). The eluted fraction was diluted 3 times with 100 mM sodium phosphate pH 7.0 and incubated with SP sepharose fast flow beads (GE Healthcare). Beads were washed 3 times with wash buffer containing 50 mM sodium phosphate pH 7.0, 100 mM NaCl, 10% glycerol, 0.01% NP40, 3 times with wash buffer 2 containing 50 mM sodium phosphate pH 7.0, 100 mM NaCl and eluted with 500 mM NaCl. The eluate fraction was concentrated and loaded onto a 4.5 mL glycerol gradient (10%-30%) and spun at 34,000 rpm in a Beckman SW60 rotor for 18 h. Fractions were collected from the bottom of the tube and protein containing fractions were pooled and dialyzed in buffer containing 50 mM Tris–HCl pH 7.5, 500 mM NaCl and 10% glycerol.

### DNA substrate preparation

The DNA substrates for enzymatic activity assays were prepared by annealing equimolar amounts of the corresponding synthetic oligonucleotides in a buffer containing 10 mM Tris–HCl (pH 7.5), 1 mM EDTA and 100 mM NaCl. DNA substrates were 5’-end labelled on the top or the bottom strand as indicated with [γ-^32^P]ATP and T4 polynucleotide kinase.

### Oligonucleotides used in this study

Underlined characters indicate the position of the mismatch. 5mC, 5hmC, 5fC, 5caC and Ø indicate 5-methylcytosine, 5-hydroxymethylcytosine, 5-formylcytosine, 5-carboxylcytosine and abasic site respectively.

TopCG: 5’ CTA ACG ATT GCC GTC GAG TAC CTA CGA GCC TGA TCG ATC GAT CGC TAA TGT CCG GCT AGA AGC GAT TCC GTA CGA TGC 3’.

BotCG: 5’ GCA TCG TAC GGA ATC GCT TCT AGC CGG ACA TTA GCG ATC GAT CGA TCA GGC TCG TAG GTA CTC GAC GGC AAT CGT TAG 3’.

BotTG: 5’ GCA TCG TAC GGA ATC GCT TCT AGC CGG ACA TTA GCG ATT GAT CGA TCA GGC TCG TAG GTA CTC GAC GGC AAT CGT TAG 3’.

Top5mCG: 5’ CTA ACG ATT GCC GTC GAG TAC CTA CGA GCC TGA TCG AT5mC GAT CGC TAA TGT CCG GCT AGA AGC GAT TCC GTA CGA TGC 3’.

Bot5mCG: 5’ GCA TCG TAC GGA ATC GCT TCT AGC CGG ACA TTA GCG AT5mC GAT CGA TCA GGC TCG TAG GTA CTC GAC GGC AAT CGT TAG 3’.

Bot5hmCG: 5’ GCA TCG TAC GGA ATC GCT TCT AGC CGG ACA TTA GCG AT5hmC GAT CGA TCA GGC TCG TAG GTA CTC GAC GGC AAT CGT TAG 3’.

Bot5fCG: 5’ GCA TCG TAC GGA ATC GCT TCT AGC CGG ACA TTA GCG AT5fC GAT CGA TCA GGC TCG TAG GTA CTC GAC GGC AAT CGT TAG 3’.

Bot5caCG: 5’ GCA TCG TAC GGA ATC GCT TCT AGC CGG ACA TTA GCG AT5caC GAT CGA TCA GGC TCG TAG GTA CTC GAC GGC AAT CGT TAG 3’.

TopCG5mC4: 5’ CTA ACG ATT GCC GT5mC GAG TAC CTA CGA GCC TGA T5mCG ATC GAT 5mCGC TAA TGT CCG GCT AGA AG5mC GAT TCC GTA CGA TGC 3’.

BotTG5mC4: 5’ GCA TCG TAC GGA AT5mC GCT TCT AGC CGG ACA TTA G5mCG ATT GAT 5mCGA TCA GGC TCG TAG GTA CT5mC GAC GGC AAT CGT TAG 3’.

TopCC: 5’ CTA ACG ATT GCC GTC GAG TAC CTA CGA GCC TGA TCG ATC CAT CGC TAA TGT CCG GCT AGA AGC GAT TCC GTA CGA TGC 3’.

TopCT: 5’ CTA ACG ATT GCC GTC GAG TAC CTA CGA GCC TGA TCG ATC TAT CGC TAA TGT CCG GCT AGA AGC GAT TCC GTA CGA TGC 3’.

TopCA: 5’ CTA ACG ATT GCC GTC GAG TAC CTA CGA GCC TGA TCG ATC AAT CGC TAA TGT CCG GCT AGA AGC GAT TCC GTA CGA TGC 3’.

BotUG: 5’ GCA TCG TAC GGA ATC GCT TCT AGC CGG ACA TTA GCG ATU GAT CGA TCA GGC TCG TAG GTA CTC GAC GGC AAT CGT TAG 3’.

BotGG: 5’ GCA TCG TAC GGA ATC GCT TCT AGC CGG ACA TTA GCG ATG GAT CGA TCA GGC TCG TAG GTA CTC GAC GGC AAT CGT TAG 3’.

BotAG: 5’ GCA TCG TAC GGA ATC GCT TCT AGC CGG ACA TTA GCG ATA GAT CGA TCA GGC TCG TAG GTA CTC GAC GGC AAT CGT TAG 3’.

BotabG: 5’ GCA TCG TAC GGA ATC GCT TCT AGC CGG ACA TTA GCG ATØ GAT CGA TCA GGC TCG TAG GTA CTC GAC GGC AAT CGT TAG 3’.

CG/GC homoduplex substrate: TopCG + BotCG; CG/GT mismatch substrate: TopCG + BotTG; CG/GT mismatch hemi-methylated substrate: TopCG + BotTG5mC4 or TopCG5mC4 + BotTG; CG/GT mismatch full-methylated substrate: TopCG5mC4 + BotTG5mC4; CG/GU mismatch substrate: TopCG + BotUG; CC/GT mismatch substrate: TopCC + BotTG; CT/GT mismatch substrate: TopCT + BotTG; CA/GC mismatch substrate: TopCA + BotCG; CC/GC mismatch substrate: TopCC + BotCG; CA/GG mismatch substrate: TopCA + BotGG; CA/GA mismatch substrate: TopCA + BotAG; CG/GG mismatch substrate: TopCG + BotGG; CG/G5mC substrate: TopCG + Bot5mCG; 5mCG/G5mC homoduplex substrate: Top5mCG + Bot5mCG; CG/G5hmC homoduplex substrate: TopCG + Bot5hmCG; 5mCG/G5hmC homoduplex substrate: Top5mCG + Bot5hmCG; CG/G5fC substrate: TopCG + Bot5fCG; 5mCG/G5fC substrate: Top5mCG + Bot5fCG; CG/G5caC substrate: TopCG + Bot5caCG; 5mCG/G5caC substrate: Top5mCG + Bot5caCG; CG/Gab substrate: TopCG + BotabG.

### Quantitative glycosylase/lyase assays

Reaction mixtures (10 µL) containing 20 mM Tris–HCl pH 7.65, 50 mM NaCl, 3 mM MgCl_2_, 5% glycerol, 1 mM DTT, 0.1 µg/µl BSA and 2 nM of end-labeled substrates was incubated for 20 min (excepted for kinetic experiment) at 37 °C with 60 nM of MBD4 (excepted as indicated). When indicated, PMS2 and MLH1 proteins were added to reaction mixture to a final concentration of 100 nM. The reaction was stopped by adding 10 µL formamide buffer (90% formamide, 10 mM EDTA, 0.1% blue bromophenol) and heating 5 min at 95 °C before loading on a 12% denaturing polyacrylamide gel. When indicated, the reaction was pre-treated with 1 µL of 1 M NaOH 10 min at 95 °C before the addition of formamide buffer. Gels were dried and quantified on a Typhoon 8600 Variable Mode Imager.

### Mapping of the nicking reaction

Enzymatic activities assays were done as described above. Products of reactions together with the products of the G + A and the C + T Maxam–Gilbert cleavage reactions performed on the same substrates were loaded on an 8% denaturing polyacrylamide gel.

## Results

### Genome-wide hypomethylation of promoters in the absence of MBD4

**Fig. 1:**
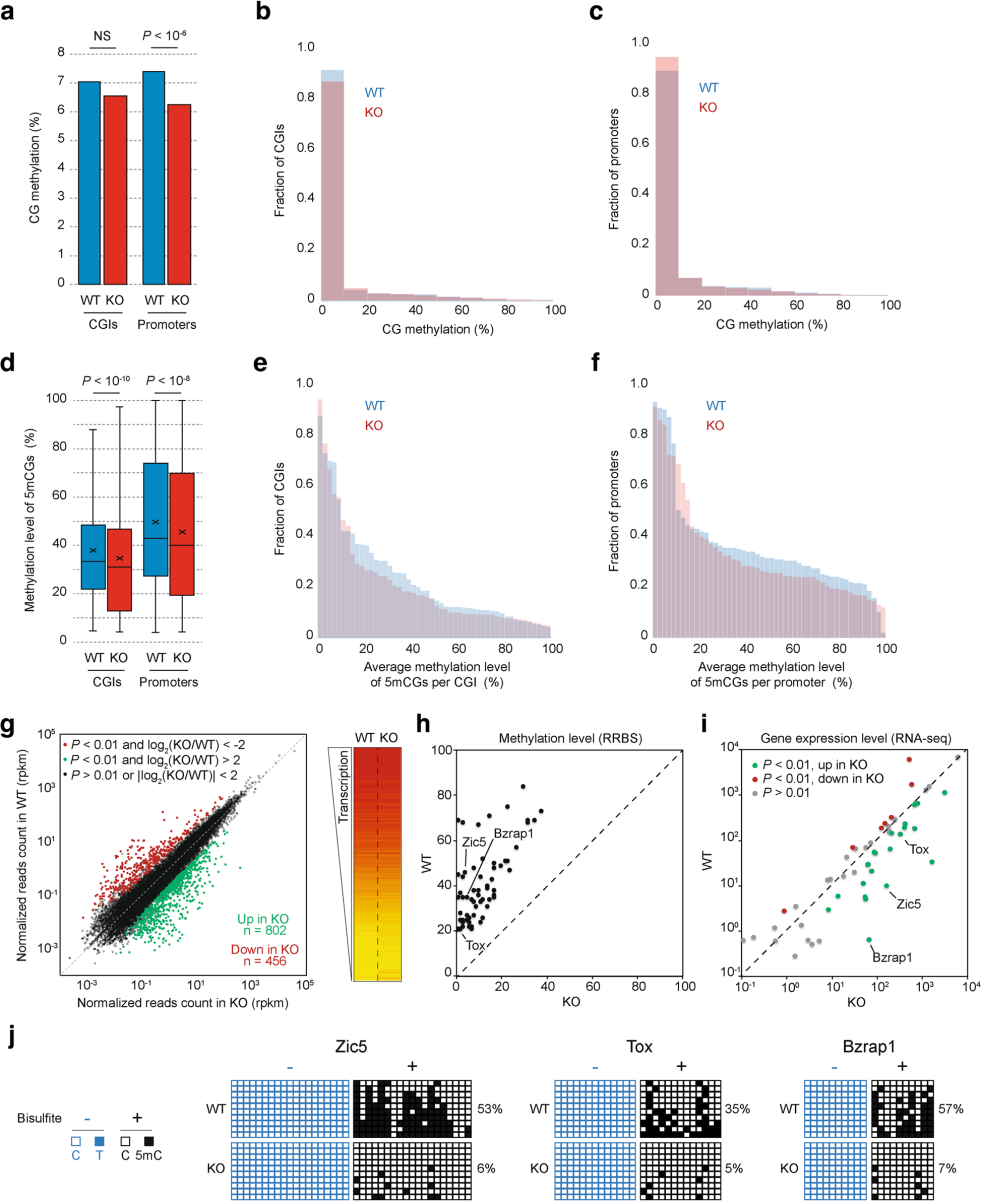
MBD4 preserves DNA methylation at promoters and maintains genes in a repressive state. **a** Average percentage of CG methylation in CGIs or in promoters for WT and KO cells. **b-c** Distribution of percentages of CG methylation per CGI (**b**) or per promoter (**c**) for WT and KO cells. **d** Average methylation level of 5mCG detected in CGIs or in promoters for WT and KO cells. **e–f** Methylation level distribution of 5mCG identified in CGIs (**e**) or in promoters (**f**) for WT and KO cells. **g** Scatter plot (left panel) and heatmap (right panel) comparing global gene expression levels between WT and KO cells. Note that 802 genes are over-expressed and 456 genes are down-regulated in the absence of MBD4 (log_2_ (KO/WT), |fc|> 2 and *P* < 0.01). **h-i** Up-regulation of demethylated promoters in the absence of MBD4. Scatter plots comparing the methylation level of differentially methylated proximal regions, determined by RRBS (**h**) and the transcription levels of corresponding genes, quantified by RNA-seq (**i**) between WT or KO MEFs for *Mbd4*. Analyses were restricted to the most significantly demethylated proximal regions in the absence of MBD4 (methylation level WT/KO > 2, number of CG > 10, *P* < 0.01, distance to nearest TSS < 5 kb). **j** Clonal standard and bisulfite sequencing showing the hypomethylation phenotype at proximal regions of *Zic5*, *Tox* and *Brzap1* genes, and revealing that the methylation loss is not accompanied by C to T transitions. The percentage of 5mCG are indicated in brackets

As the protein MBD4 can repair the product of 5mC deamination, we asked how the cell uses the specific property of this enzyme to regulate the steady state of DNA methylation level. To this end we used WT (*Mbd4*^+/+^) and KO (*Mbd4*^−/−^) primary MEFs. We first hypothesized that the absence of MBD4 would generate alterations in gene promoter methylation patterns, which would in turn affect their transcriptional status. DNA methylation distribution was analyzed by reduced representation bisulfite sequencing (RRBS) [53]. RRBS provides single-nucleotide resolution and quantitative DNA methylation measurements for the majority of CG-rich regions such as CGIs and promoters [54]. Summary of the data quantity after each step of filtration is shown in Supplementary Fig. 1a and both samples showed near complete bisulfite conversion of non-CpG cytosines (> 99%). Since only half of all CGIs overlap with TSSs despite the fact that more than 70% of annotated gene promoters are associated with a CGI [55], we decided to analyze separately promoters from CGIs (Supplementary Fig. 1b). Our data covered 77% to 87% of cytosines theoretically covered by RRBS in both CGI and promoter regions for WT and KO cells (Supplementary Fig. 1c). Since approximately 95% of the detected 5mC were found to occur in CpG context (Supplementary Fig. 1d), we decided to focus our study only on 5mCG. The methylation level of CGIs and promoters was assessed by two different parameters: the percentage of methylated CpGs per region and the methylation level of each 5mCG. In agreement with previously published data [56], CGIs and promoters were found to be poorly methylated (6.3–7.4% of 5mCG) with little or no difference between WT and KO cells (Fig. 1a-c and Supplementary Tables 1–4). Importantly, the methylation levels of 5mCG in both CGIs and promoters significantly decreased in the absence of MBD4 (*P* < 10^–10^ and *P* < 10^–8^, respectively; Fig. 1d). For a significant portion of 5mCGs that were originally methylated over 20% in WT cells, their methylation levels dropped to less than 10% in KO cells (Fig. 1e-f and Supplementary Tables 1–4). These results strongly indicate that, although the number of methylated CpGs only modestly decreased in KO cells (Fig. 1a-c), their methylation levels significantly decreased in the absence of MBD4 (Fig. 1d-f). It is reasonable to conclude that MBD4 plays a protective role in preventing the demethylation of methylated CGs. Notably, the loss of methylation primarily affected promoters and CGIs with low methylation levels (Supplementary Fig. 1d-e).

We next investigated how DNA methylation defect affects gene expression. Genome-wide transcriptome analysis of *Mbd4*^−/−^ cells identifies a total of 1,258 genes (with *P* < 0.01 and ∣log_2_ fold change∣ > 2)) having strong transcriptional de-regulation compared to the control *Mbd4*^+/+^ cells where 802 of these genes were found up-regulated and 456 were found down-regulated (Fig. 1g and Supplementary Table 5). We hypothesized that the hypomethylated CGI promoters are up-regulated. To test this, we selected genes containing the most significantly demethylated proximal region (methylation level WT/KO > 2, number of CpG > 10, *P* < 0.01, distance to nearest TSS < 5 kb) and correlated their methylation level (Fig. 1h) to their transcriptional states (Fig. 1i). Accordingly, around 40% of the hypomethylated CGI promoters are transcriptionally up-regulated (*P* < 0.01) in the absence of MBD4. Clonal standard and bisulfite sequencing further confirmed the hypomethylation phenotype at proximal regions of *Zic5*, *Tox* and *Brzap1* genes, and revealed that the methylation loss is not accompanied by C > T transitions (Fig. 1j).

These data, taken as a whole, illustrate that MBD4 is required for both preserving the methylation status of its target genes and maintaining them in a repressive state.

### Loss of MBD4 leads to retro-elements derepression

**Fig. 2:**
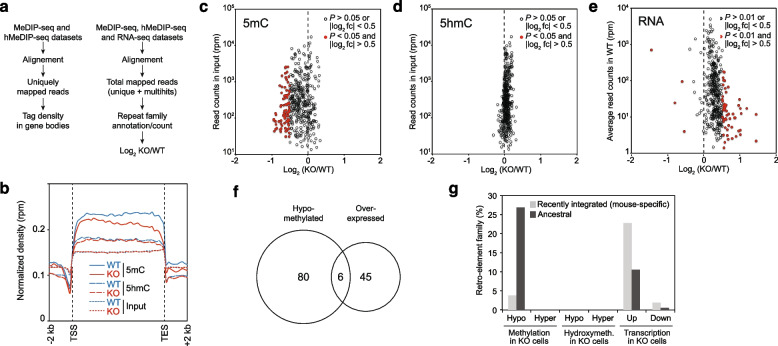
Demethylation and derepression of retrotransposons in the absence of MBD4 in MEFs. **a** Flowchart of computational analyses used in this study. **b** Normalized 5mC and 5hmC read densities across gene bodies in WT and KO MEFs for *Mbd4*. **c-d** Scatter plots representing the relative fold change (log_2_-ratio KO/WT) for 5mC (**c**) and 5hmC (**d**) enrichment at each retroelement family in function of their relative abundance in the mouse genome (read counts from input sample are normalized in reads per million, rpm) in the absence of MBD4. **e** Scatter plot representing the relative fold change (log_2_-ratio KO/WT) in the expression of each retrotransposon family in function of their relative expression in the absence of MBD4 (average read counts in WT samples normalized in reads per million, rpm). **f** Venn diagram showing the overlap between hypomethylated and over-expressed retrotransposon families in the absence of MBD4. **g** Percentage of retrotransposon families (mouse specific *vs* ancestral) showing a statistically significant change in cytosine modification enrichment (|log_2_ fold change|> 0.5 and *P* < 0.05) or in their expression (|log_2_ fold change|> 0.5 and *P* < 0.01) in the absence of MBD4 in MEFs

Our RRBS data reveal methylation loss at CpG-rich regions exhibiting low methylation levels in *Mbd4*^−/−^ cells. But the vast majority of the mouse genome is highly methylated and CpG-poor, such as repetitive elements that make up 40% of the genome. We then decided to extend our analysis to the globally methylated and CpG-poor genomic landscape by using DNA immunoprecipitation sequencing (DIP-seq). We have carried out a genome-wide comparative analysis of the 5mC (MeDIP-seq) and 5hmC (hMeDIP-seq) patterns in both MEFs WT and KO for *Mbd4*. We first analyzed the DNA methylation/oxidation pattern of gene bodies by analyzing uniquely mapped reads (Fig. 2a, left panel). Tag densities were calculated in 100 bp sliding windows spanning ± 2 kb of gene bodies using datasets normalized to the total number of unique reads. Interestingly, we observed a strong decrease in 5mC density across genes peaking at the 3’ end of gene bodies. In contrast, no difference was detected by analyzing 5hmC or input reads. These data show that MBD4 is also required to maintain DNA methylation in gene bodies.

A DIP-seq approach allows a high coverage of repetitive elements [57]. In order to highlight a potential function of MBD4 in retro-element repression, we extended our initial DIP-seq and RNA-seq analyses to include “unmappable” multihit reads (Fig. 2b, right panel). The unique reads can be mapped to specific genomic sites while the multihit reads, which represent repetitive elements [58], can only be assigned to their specific repeat family. Reads were aligned to repetitive elements in two passes (see materials and methods for details). The fold change in read counts between MEFs WT and KO for *Mbd4* were calculated for each DNA repeat family, and represented as log_2_(KO/WT) ratio (Fig. 2c, d and e for MeDIP-seq, hMeDIP-seq and RNA-seq, respectively). We restricted our analysis to retrotransposon families (including long terminal repeats (LTR), long (LINE) and short (SINE) interspersed nuclear elements), which account for more than 90% of the repetitive elements of the mouse genome. While we did not detect any change in 5hmC enrichment at retro-elements in *Mbd4*^−/−^ cells (Fig. 2d), we observed a global loss of 5mC affecting significantly 20% (86/410, *P* < 0.05 and |log_2_ fold change|> 0.5) of the covered families (Fig. 2e). Importantly, the hypomethylation phenotype was accompanied by a global reactivation of retrotransposons affecting significantly 14% (56/410, *P* < 0.01 and |log_2_ fold change|> 0.5) of all families. However, we only found six families that were both hypomethylated and reactivated (Fig. 2f). To better understand the limited correlation between the methylome and transcriptome, we conducted separate analyses for SINE (Supplementary Fig. 2a), LINE (Supplementary Fig. 2b), and LTR (Supplementary Fig. 2c-d) elements based on their evolutionary age. This approach enabled us to distinguish Mbd4 deficiency-dependent effects on lineage-specific (mouse) and ancestral retro-element families (Fig. 2g and Supplementary Fig. 2a-d). While the methylation loss was mainly detected for the oldest subfamilies (4% and 27% of the mouse-specific and ancestral retro-element subfamilies, respectively), the transcriptional derepression was more pronounced for the youngest subfamilies (23% and 11% of the mouse-specific and ancestral retro-element subfamilies, respectively). We attributed this apparent discrepancy to different CpG content, which is twofold higher in the youngest retro-elements than in the oldest one (Supplementary Fig. 2). MeDIP-seq data being strongly dependent to the local density of 5mC, and therefore to CpG content, we postulate that a moderate decrease in 5mC density is more detectable at CpG-poor retro-elements. Conversely, we hypothesized that MBD4 proteins are more prevalent at CpG-rich elements, making these regions transcriptionally more susceptible to MBD4 depletion and potentially subject to reactivation through both DNA methylation-dependent and -independent mechanisms. To conclude, our DIP-seq analysis reveal that the MBD4 function in DNA methylation maintenance is not restricted to punctuated and weakly methylated CpG-rich regions (as shown by single base resolution RRBS approach), but is also extended to the globally methylated CpG-poor genomic landscape.

### MBD4 is associated in vivo with core MMR proteins

**Fig. 3:**
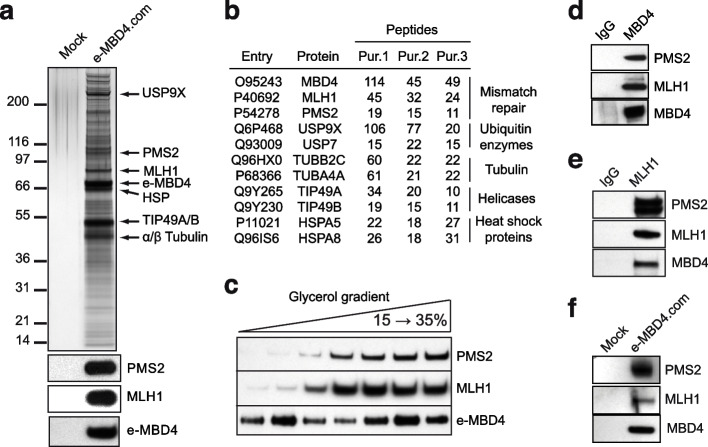
MBD4 interacts with mismatch repair proteins in vivo. **a** MBD4 complex (e-MBD4.com) was purified by double immunoaffinity from an HeLa cell line stably expressing MBD4 fused with N-terminal Flag- and HA-epitope tags (e-MBD4) and was run on an SDS PAGE. Silver staining of the SDS gel (top panel) and immunoblotting detection (bottom panel) of the proteins associated with e-MBD4 are shown. **b** The major polypeptides detected by mass spectrometry analysis of three independent e-MBD4.com purifications. **c** Western blot analysis of the e-MBD4 complex separated by glycerol gradient fractionation. **d** Endogenous MBD4 specifically co-precipates with MLH1 and PMS2. **e** Endogenous MLH1 co-precipates with MBD4 and PMS2. **f** Western blot analysis of e-MBD4.com purified by double immunoaffinity from MEFs

In order to decipher the molecular mechanisms implicated in MBD4-dependent DNA methylation maintenance in vivo, we sought to study the enzymatic properties of MBD4. The properties of purified recombinant MBD4 have previously been analyzed and the reported data suggests that the recombinant protein exhibits G/T and G/U mismatch specific monofunctional glycosylase activity [27, 28, 59, 60]. The native MBD4 complex could, however, have features distinct from those of the MBD4 protein alone. To test this, we purified the epitope-tagged MBD4 complex (e-MBD4.com) from HeLa cells stably expressing hemagglutinin (HA) and FLAG epitope tagged MBD4 (Fig. 3a). MBD4 together with the DNA helicases TIP49A/B and the MMR proteins (MLH1 and PMS2) were identified as major components of e-MBD4.com by both mass spectrometry (Fig. 3b and Supplementary Table 10) and Western blotting (Fig. 3a, lower panel). Fractionation of the e-MBD4 complex on a glycerol gradient confirmed that MLH1 and PMS2 are stable components of this complex (Fig. 3c). This interaction was further validated by immunoprecipitating either the endogenous MBD4 (Fig. 3d) or MLH1 (Fig. 3e) complexes from non-tagged HeLa cell nuclear extracts using specific antibodies. We next analyzed the composition of the MBD4 complex in mouse embryonic fibroblast (MEF) cell lines stably expressing e-MBD4. Western blotting demonstrates that the MEF e-MBD4 complex exhibits the same composition as the e-MBD4 complex isolated from HeLa cells and thus, it should mechanistically function in the same way (Fig. 3f). We conclude that MBD4 forms in vivo a complex with the PMS2/MLH1 heterodimer.

### The MBD4 complex shows methyl-directed G/T mismatch specific endonuclease activity

Some common glycosylases are known to exhibit an endonuclease mismatch activity [61, 62]. To test if this is the case for the e-MBD4.com, we carried out nuclease assays on substrate DNA containing different types of mismatches. The data clearly show that: (i) the e-MBD4 complex is able to cleave the G/T (or G/U) mismatch (Supplementary Fig. 3a-b), but not the cytosine, the methyl-, the hydroxymethyl-, the formyl- or the carboxyl-cytosine substrates (Supplementary Fig. 3c), and (ii) the cleavage is achieved at the abasic site on the “T”-containing strand of a G/T mismatch substrate (Supplementary Fig. 3a). Importantly, no NaOH treatment of the reaction products was needed for generation of the cleavage products (Supplementary Fig. 3a). Therefore, the e-MBD4.com exhibits G/T mismatch specific endonuclease activity.

**Fig. 4:**
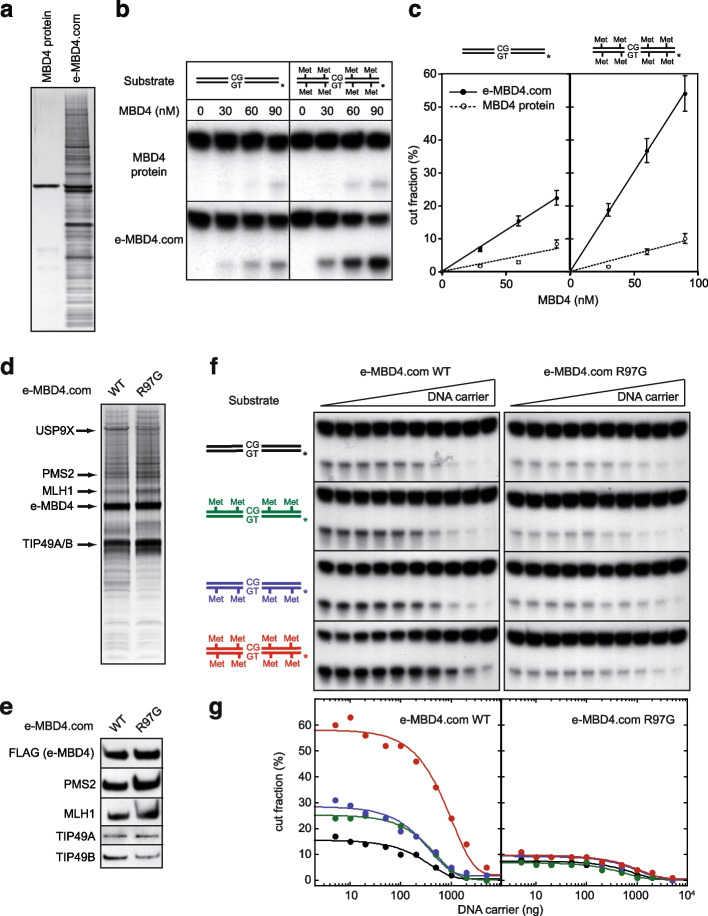
MBD4 is a methyl-directed mismatch endonuclease. **a** SDS PAGE silver staining of purified e-MBD4 and e-MBD4-com. **b** Unmethylated or full-methylated G/T mismatch containing substrates were incubated with increasing amounts of recombinant MBD4 protein or with e-MBD4.com and analyzed as described in Supplementary Fig. 3. The reaction products were not treated with NaOH. **c** Quantification of the data presented in (**b**). The means of three independent experiments are shown. **d-e** Single point mutation at R97G was introduced in the coding sequence of e-MBD4 to generate a dead methyl binding MBD4 mutant (e-MBD4 R97G) and HeLa cell lines stably expressing e-MBD4 R97G were generated. SDS PAGE gel silver staining (**d**) and immunobloting analysis (**e**) of e-MBD4.com (WT) and e-MBD4.com R97G complexes. **f** Nucleases assays for e-MBD4.com (WT) and e-MBD4.com R97G. Full-methylated (red), hemi-methylated (blue and green) or un-methylated (black) G/T mismatch containing substrates were incubated in the presence of either e-MBD4.com (WT) or e-MBD4.com R97G and increasing amounts of carrier DNA. The experiments were carried out as described Supplementary Fig. 3. The reaction products were not treated with NaOH. **g** Quantification of the results presented in (**f**). Red and black curves correspond to full- and unmethylated G/T containing substrates, respectively. Green and blue curves correspond to hemi-methylated substrates

To analyze whether the e-MBD4.com endonuclease activity was dependent on the methylation status of the substrate, we carried out similar experiments, but with fully methylated (on both strands) G/T mismatch containing substrates by using identical amounts of either highly purified MBD4 alone (Fig. 4a) or MBD4 in the context of the e-MBD4.com. The purified MBD4 protein was able to induce some weak, non-methylation dependent cleavage of the substrate (Fig. 4b, upper panel and Fig. 4c). The e-MBD4.com shows ~ threefold higher activity for unmethylated DNA relative to that of the MBD4 protein (Fig. 4b-c). The e-MBD4.com endonuclease activity was, however, strongly methylation dependent and ~ 8–tenfold higher e-MBD4.com induced cleavage (compared to this for the MBD4 protein) for fully methylated substrates was measured (Fig. 4b-c). These data indicate that the association of MBD4 with its partners modulates its enzymatic properties and, as a result, MBD4 acquires a much higher G/T specific endonuclease activity, which isdependent on the methylation of the DNA substrate.

We next addressed the role of the methyl binding domain of MBD4 by generating a stable HeLa cell line expressing R97G mutated e-MBD4 (the substitution of R97 with G results in a dead methyl binding domain [18]). Both silver staining (Fig. 4d) and Western blotting (Fig. 4e) showed that the composition of the purified methyl binding dead e-MBD4 complex (e-MBD4.com R97G) is identical to the native e-MBD4.com. Nuclease assays revealed that the R97G mutant e-MBD4.com does not discriminate between unmethylated, hemi-methylated and fully methylated mismatch-containing substrates. In all cases, cleavage in the absence of carrier DNA is very low (~ 10%) and, with increasing concentrations of carrier DNA, this activity progressively decreases (Fig. 4f-g, right panels). In contrast, the native complex (e-MBD4.com WT) discriminates clearly between methylated and unmethylated substrates and its cleavage efficiency, compared to the mutant e-MBD4.com R97G, is much higher at the respective carrier DNA concentrations (Fig. 4f-g, left panels). In particular, dramatic differences in cleavage efficiencies for both complexes are measured for the fully methylated substrates (Fig. 4f-g). These results demonstrate that the e-MBD4.com endonuclease activity strongly depends on the degree of methylation of the DNA substrate and that the methyl-binding domain of MBD4 targets the MBD4-MMR complex on methylated DNA.

### MBD4 is an endonuclease with a bifunctional glycosylase/AP lyase activity

**Fig. 5:**
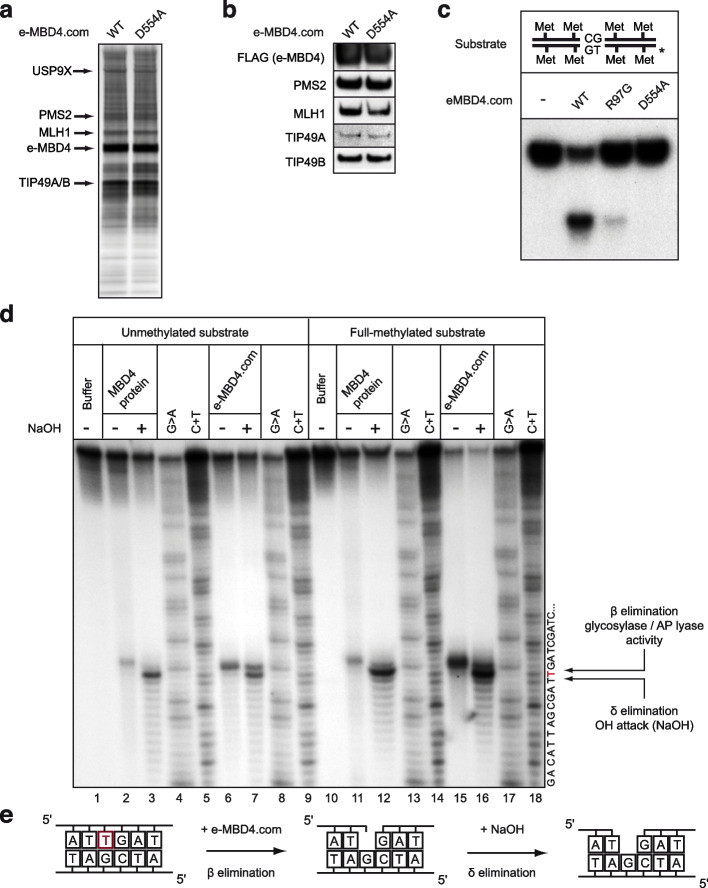
MBD4 is a bifunctional DNA glycosylase/AP lyase enzyme. **a-b** HeLa cell lines stably expressing the e-MBD4 glycosylase dead mutant (e-MBD4 D554A) were used to purify by double immunoaffinity the e-MBD4 D554A complex. Silver staining of the SDS PAGE gel (**a**) and immunoblotting (**b**) for e-MBD4.com (WT) and the mutant e-MBD4 D554A.com. **c** Nuclease assay for e-MBD4.com (WT) and both mutant e-MBD4.com R97G and e-MBD4 D554A.com. The experiments were carried out as described in Figure EV3A. The reaction products were not treated with NaOH. **d** Unmethylated and full-methylated G/T mismatch substrates were incubated with MBD4 protein or with e-MBD4 complex as described in Supplementary Fig. 3. Both reaction products as well as the products of Maxam&Gilbert sequencing assay were separated on PAGE under denaturing conditions. The « T» in red indicates the migration of the respective cleavage « T» product obtained by the Maxam & Gilbert sequencing reaction. **e** Schematics of the bi-functional glycosylase/AP-lyase activity of MBD4

We next asked whether the MBD4 glycosylase domain is important for the observed G/T specific endonuclease activity. To this end we substituted amino acid residue D554 with alanine (A) and created a glycosylase dead mutant e-MBD4 protein [63]. Then, we established stable HeLa cell lines expressing the e-MBD4 D554A mutant. Both protein gel analysis and Western blotting show that the glycosylase dead mutant e-MBD4 D554A complex purified from the stable HeLa cell lines has a protein composition identical to the WT e-MBD4 complex (Fig. 5a-b). The mutant e-MBD4.com D554A exhibits, however, no endonuclease activity (Fig. 5c). We conclude that the activity of the MBD4 glycosylase domain is required for its endonuclease activity.

Past studies have demonstrated that MBD4 possesses G/T specific glycosylase activity [27, 28, 60]. Several glycosylases show, however, also AP lyase activity, i.e., the excision of the base is followed by AP lyase-mediated cleavage of the phosphate backbone at the abasic site [61, 62]. If the AP lyase product is generated through β elimination, it retains the abasic residue at its 3’ end (Fig. 5e and [64, 65]). This product migrates slower on sequencing PAGE than the respective product obtained from the Maxam & Gilbert sequencing reaction. Upon treatment of such AP-lyase generated products with NaOH, the phospho-diester bond is cleaved (δ elimination), the abasic residue is released and the mobility of the resulting product becomes identical to that generated by the Maxam & Gilbert sequencing reaction.

Our data suggest that MBD4 possesses an AP-lyase type endonuclease activity. To test this, we have used the above-described procedure (Fig. 5d-e). Treatment with NaOH of either MBD4 or e-MBD4.com reaction products resulted in clear increase of their migration rate in PAGE under denaturing conditions. The migration position of the NaOH treated products was identical to that of the products of the Maxam & Gilbert sequencing reaction obtained upon cleavage at the respective thymine (Fig. 5d). These observations reveal that MBD4 possesses an AP-lyase endonuclease activity, which operates through β elimination (Fig. 5e).

### The methyl-directed nuclease activity of MBD4 is dependent on its physical interaction with MLH1

**Fig. 6:**
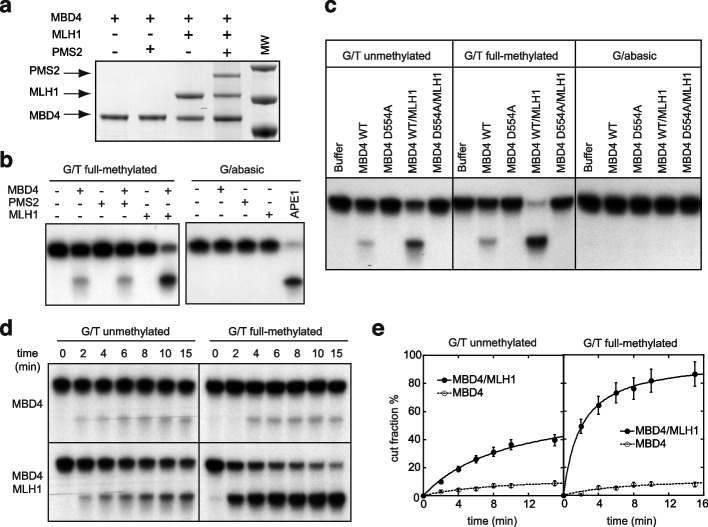
The methyl-directed nuclease activity of MBD4 is dependent on its physical interaction with MLH1. **a** SDS PAGE coomassie staining of the purified MBD4 protein, MBD4/MLH1 and MBD4/MLH1/PMS2 complexes co-expressed in the baculovirus system. **b** Nuclease assays. The indicated combinations of recombinant proteins were mixed with methylated G/T or abasic-containing substrates and the cleavage reaction was carried out and analyzed as described in Figure EV3A. The reaction products were not treated with NaOH. **c** Nuclease assays using either the recombinant MBD4 protein or the purified MBD4/MLH1 complex on un-methylated substrates (left panel), full-methylated substrates (middle panel), or abasic site-containing substrates (right panel). Note that any activity was detected with substrates containing an abasic site excluding the presence of nuclease contaminant. **d** Un-methylated or full-methylated substrates were incubated with identical amount of either MBD4 alone (upper panel) or MBD4 in complex with MLH1 (lower panel) for the indicated times and analyzed as described in Supplementary Fig. 3. The reaction products were not treated with NaOH. **e** Quantification of the data presented in (**d**). The means of three independent experiments are shown

Having demonstrated the AP-lyase endonuclease activity of MBD4, we next sought to analyze the role of the MMR proteins in the regulation of this newly identified activity. We have expressed either separately or co-expressed together the recombinant MBD4, MLH1 and PMS2 proteins in the baculovirus system. We were able to reconstitute the MBD4/MLH1 and the MBD4/MLH1/PMS2 complexes but not the MBD4/PMS2 complex, suggesting a direct physical interaction between MBD4 and MLH1 (Fig. 6a). Reconstitution of the in vitro nuclease assay using individual recombinant proteins shows that MBD4, but not the MMR proteins, has some weak endonuclease activity (Fig. 6b), a result in agreement with the data presented in Fig. 4b and excluding a potential enzymatic function of MMR proteins in e-MBD4.com endonuclease activity. Remarkably, the presence of MLH1 results in a dramatic increase of the endonuclease activity of MBD4 with marked preference for fully methylated substrates (Fig. 6b-c). Note that the enzymatic dead MBD4 D554A mutant protein alone, or complexed with MLH1, did not exhibit endonuclease activity (Fig. 6c). Similarly, none of the purified recombinant proteins exhibited endonuclease activity towards a DNA substrate containing abasic sites (cleavable by the recombinant apurinic/apyrimidinic endonuclease APE1), ruling out the presence of a non-specifically associated contaminating AP-lyase activity (Fig. 6b, right panel and Fig. 6c, right panel). All together, these data reveal that the recombinant MLH1 alone and not some biochemically undetectable contaminant is responsible for stimulating the MBD4 endonuclease activity. The nuclease time-course assay (Fig. 6d-e) shows that the purified MBD4/MLH1 complex cuts much more efficiently the G/T mismatch substrate compared to MBD4 alone. The most drastic differences in the cleavage efficiency are measured for fully methylated substrates, where the cleavage kinetics (as assessed by the initial slope of the nuclease assay time course curve) of the MBD4/MLH1 is at least tenfold higher than for MBD4 alone (Fig. 6d-e). Therefore, by using recombinant components we have been able to reconstitute in vitro the enzymatic properties of the isolated native MBD4 complex and demonstrate the crucial role that MLH1 plays in the MBD4 complex endonuclease activity.

## Discussion

The described in vitro data demonstrate that MBD4 is an unusual glycosylase having two domains essential for its functions. In addition to its glycosylase activity, the MBD4 catalytic domain exhibits an AP lyase activity. These two activities are required for both the removal of the thymine base and cleavage of the DNA phosphate backbone. In cells, MBD4 forms a complex with the MMR proteins MLH1 and PMS2 as well as with other proteins. This MBD4-MMR protein complex possesses a much higher cleavage efficiency than MBD4 alone. Experiments with highly purified recombinant proteins show the MMR protein MLH1 is required for this effect. Similar “boosting” function for MLH1 has already been observed for EXO1 and PMS1 proteins, two nucleases implicated in MMR pathway in eukaryotes. Indeed, the physical interaction between MLH1 and EXO1 is required for the endonuclease function of EXO1 in the MMR pathway [66]. A recent structural study has also revealed that the highly conserved C terminus of MLH1 forms part of the PMS1 endonuclease site [67]. Together, these data define MLH1 as a nuclease effector protein. In addition, the MBD4 complex has a clear preference for methylated G/T mismatch containing substrates, which is determined by its methyl-binding domain (our data). Therefore, MBD4 appears to be specifically designed to repair G/T mismatches in the vicinity of methylated CpGs.

The absence of MBD4 in primary MEFs leads to substantial methylation loss affecting CGI promoters, gene bodies, and repetitive elements with low CpG density. Our RRBS analysis identified only 51 significantly hypomethylated promoters following MBD4 loss. This number is relatively low compared to the 802 genes found significantly overexpressed without MBD4. Given that 90% of these promoters are already poorly methylated or unmethylated in WT cells (% CG methylation < 10%), it is not surprising that only a small proportion of them exhibit significant hypomethylation. We hypothesize that MBD4 depletion results in the overexpression of a significant portion of genes through DNA methylation-independent mechanisms, such as impaired recruitment of co-repressor complexes to methylated DNA, or other alternative indirect effects.

**Fig. 7:**
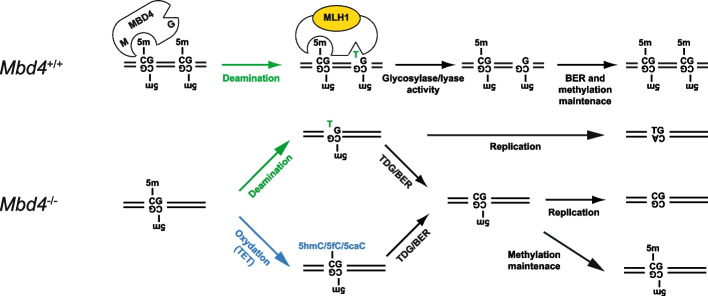
Model for the function of MBD4 in vertebrates. MBD4 bind 5mC through its methyl-binding domain (M). If a spontaneous deamination conversion of 5mC to T occurs, MLH1 activates MBD4, which, through the glycosylase/AP lyase activity of its glycosylase domain (G), removes the thymine base and cleaves the phosphate backbone. The generated abasic 3’ cleaved site is then repaired by BER. Finally, the methylation mark is restored through the action of the DNA methylation maintenance pathway. In the absence of MBD4, 5mC are more susceptible to deamination leading to the formation of G/T mismatch and/or to oxidation by TET enzymes to produce 5hmC, 5fC and 5caC. G/T mismatch, 5fC and 5caC can be processed by TDG and the BER machinery, the methylation mark is then restored by the DNA methylation maintenance pathway. If a round of replication takes place before the action of TDG and/or of the DNA methylation maintenance machinery, the 5mC mark can be lost

Whereas the hypomethylation of repetitive elements appears to be a hallmark of cancer cells [68–70], how this is related to cancer development is poorly understood. The present study reveals that MBD4 preserves the 5mC marks at retro-elements in MEFs, and then is implicated in their transcriptional silencing. Of note, we did not detect any change in 5hmC densities neither at genes nor at repetitive elements in *Mbd4*^−/−^ cells. This result indicates that the TET-mediated oxidation pathway should not be implicated in the genome-wide methylation loss observed in absence of MBD4, which could be either due to deamination events or failure of the MMR mutant complex to recognize C to T transitions. Nevertheless, we cannot rule out that in absence of MBD4, some 5mC undergo successive steps of oxidation leading to the formation of 5fC and 5caC. In both scenarios, G/T mismatch, 5fC or 5caC would be excised and repaired to regenerate unmodified cytosines by the concerted action of thymine-DNA glycosylase (TDG) and the base excision repair (BER) enzymes (Fig. 7). Accordingly, we showed that the hypomethylation phenotype at the proximal region of *Zic5*, *Tox* and *Bzrap1,* three genes up-regulated in *Mbd4*^−/−^, are not accompanied by C to T transitions.

During evolution, the appearance of MBD4 protein seems to have coincided with the vertebrate lineage establishment [71]. This event parallels the transition from mosaic to global DNA methylation of the genomes, and consequently would reflect the onset of a CpG-poor genomic landscape due to spontaneous deamination of 5mC and its transition into T. To protect the essential for cell life methylation status of vertebrate genomes, evolution has created MBD4, a puzzling enzyme containing two different domains: one able to recognize the methylation substrate (i.e. the 5mC through its methyl-binding domain) and the other one the product of its deamination (i.e. the G/T mismatch through its glycosylase domain). This makes the function of MBD4 unique within the MBD class of proteins and stresses the role of its glycosylase domain in preserving DNA methylation. This is in contrast to the genes associated with other MBD proteins (MBD1, MBD2 or MeCP2), where the siRNA depletion of these proteins resulted only in derepression of the respective genes and not in demethylation of their promoters [72–77].

We propose the following simplistic model for the function of MBD4 (Fig. 7). MBD4 is bound through its MBD to 5mC. In this way, MBD4, either by steric hindrance or/and by recruiting repressive complexes, maintain chromatin in a repressive state. As a result of spontaneous deamination, the 5mC is mutated to T and thus, a G/T mismatch is formed. MBD4 excises the T via its glycosylase/AP lyase activity and the BER machinery further repairs the “gap”. Subsequent methylation of the repaired CpG allows the binding of another MBD4 molecule to the methylated dinucleotide though its MBD, and thus, the repressive state is preserved. If MBD4 is absent, the T-G mismatch cannot be repaired efficiently and the methylation mark is lost as observed in *Mbd4*^−/−^ cells. In addition, in the absence of MBD4, C to T transitions at CpG sites will be generated which would lead to genome instability, as determined in *Mbd4*^−/−^ mice [32, 33] and several human cancers [34–36]. Considering that spontaneous deamination rates are extremely low [78], we cannot exclude that a significant portion of C to T transitions may result from 5mC being incorrectly paired with A during replication and not efficiently repaired in MMR mutants [79].

### Supplementary Information

**Additional file 1: Supplementary Figure 1.** Hypomethylation of promoters in the absence of MBD4 in MEFs. (a) Table summarizing the data obtained by RRBS after filtering and alignment of the raw reads. (b) Venn diagram showing the overlap between the two databases analyzed in this study. RRBS covered CGI and promoter databases correspond to databases described in methods but restricted to elements targeted by restriction enzyme digestion (14,416 and 15,710 elements, for CGIs and promoters respectively, which correspond to 90 % and 68 % of the corresponding mouse database). (c) Table listing the covered number of cytosines in each sequence context (CG, CHG and CHH, H represents non-G base). Theoretical values indicate cytosines located in theoretical enzyme cutting regions, and experimental values are the actual number of cytosines covered by sequencing reads. (d) Percentage of methylcytosines identified in CGIs or in promoters for WT and KO cells in each sequence context. (e-f) Dot blots representing the average methylation level of 5mCG as a function of the percentage of CG methylated per each CGI (d) or per each promoter (e) for WT (left panels) and KO (right panels) cells.

**Additional file 2: Supplementary Figure 2.** Genome-wide demethylation and derepression of retrotransposons in *Mbd4*^-/-^ MEFs. (a**-**d) Fold change (log_2_ ratio KO/WT) in 5mC enrichment or in the expression of SINE (a), LINE (b) and LTR (c-d) subfamilies in absence of MBD4 in MEFs. SINE and LINE subfamilies were arranged from the youngest to the oldest subfamilies to distinguish between lineage-specific (mouse) and ancestral families. Within the different LTR families, RMSK database distinguishes retro-elements corresponding to external domains (LTRext, containing the regulatory regions of the LTR) from those corresponding to internal domains (LTRint, containing the coding sequences of the proteins, necessary for the life cycle of the integrated viruses). Bearing this in mind, we carried out independent analyses for these two regions LTRext (c) and LTRint (d). LTR subfamilies were then sorted by classes (ERV1, ERVK and ERVL), and within each class of LTR, young mouse-specific LTR subfamilies were isolated from ancestral families. Asterisks (*) indicate significant difference (|log_2_ ratio fold change| > 0.5 and *P *< 0.05). (e) Average CG density (number of CG dinucleotides per 100 bp) of lineage-specific (mouse) and ancestral retro-elements in the mouse genome.

**Additional file 3: Supplementary Figure 3.** The MBD4 complex exhibits G/T mismatch specific endonuclease activity. (a) *In vitro* glycosylase/nuclease assays. e-MBD4.com was mixed with the indicated substrates (* indicates the labeled strand), incubated for 20 minutes at 37°C and the products of the reaction were run on PAGE under denaturing conditions. Note that the generation of cut products does not require NaOH treatment. (b**-**c) e-MBD4.com were incubated with indicated substrates (* indicates the labeled strand) as described in (a). Reaction products were not treated with NaOH.

**Additional file 4: Supplementary Tables 1 and 2.** Methylation information for CGIs in MEFs WT (1) or KO (2) for *Mbd4*. These tables list each mouse RRBS covered CGI with the following informatio: genomic annotation, distance to nearest TSS, name of nearest gene, CG density, coverage depth, percentage of C methylated and average methylation level of 5mC at each sequence context (CG, CHG, CHG, H represents non-G base).

**Additional file 5: Supplementary Tables 3 and 4.** Methylation information for promoters in MEFs WT (3) or KO (4) for *Mbd4*. These tables list each mouse RRBS covered promoter with the following information: genomic annotation, gene name, coverage depth, percentage of C methylated and average methylation level of 5mC in each sequence context (CG, CHG, CHG, H represents non-G base).

**Additional file 6: Supplementary Table 5.** Listing of the most significantly deregulated genes (|fc|>2 and *P *< 0.01) in absence of MBD4. The following information are indicated for each deregulated gene: Ensemble gene id, raw read counts (KOrep1, KOrep2, WTrep1, WTrep2), normalized read counts (KOrep1, KOrep2, WTrep1, WTrep2), fold change (log_2_-ratio KO/WT), *p*-value, *p*-value adjusted for multi-testing, gene name, gene description.

**Additional file 7: Supplementary Table 6.** Listing of the most significantly differentially methylated regions (DMR, *P *< 0.01) between MEFs WT and KO for *Mbd4*. The following information are indicated for each DMR: genomic annotation, methylation rate (KO, WT, WT/KO) and variation (KO, WT), coverage depth and variation (KO, WT), number of CpG, number of uncovered CpG (KO, WT).

**Additional file 8: Supplementary Tables 7 and 8.** Repeat analyses of MeDIP-seq (7) and hMeDIP-seq (8) datasets. These tables list each mouse repeat family with the following informations: Repeat-Masker family name, raw read counts (input-WT, input-KO, IP-WT, IP-KO), normalized read counts (input-WT, input-KO, IP-WT, IP-KO), log_2_-ratio (KO/WT).

**Additional file 9: Supplementary Table 9.** Genome-wide transcriptome analyses of repetitive elements. This table list each mouse repeat family with the following informations: Repeat-Masker family name, raw read counts (KOrep1, KOrep2, WTrep1, WTrep2), normalized read counts (KOrep1, KOrep2, WTrep1, WTrep2), base mean, log_2_-ratio (KO/WT), *p*-value, *p*-value adjusted for multi-testing.

**Additional file 10: Supplementary Table 10.** Full list of peptides and proteins identified by mass spectrometry analysis of the epitope-tagged MBD4 complex purified from HeLa cells by double immunoaffinity.

## Data Availability

Raw datasets (RNA-seq, RRBS, MeDIP-seq and hMeDIP-seq), processed expression datasets (RNA-seq) and processed methylation datasets (MeDIP-seq, hMeDIP-seq and RRBS) obtained in WT or KO MEFs for *Mbd4* have been deposited in the Gene Expression Omnibus (GEO) under the accession number GSE66397.
