# Supplementary figures and images for: MBD4 loss results in global reactivation of promoters and retroelements with low methylated CpG density
Source: J Exp Clin Cancer Res. 2023 Nov 14;42:301. doi: 10.1186/s13046-023-02882-z (PMC10644448; doi:10.1186/s13046-023-02882-z)

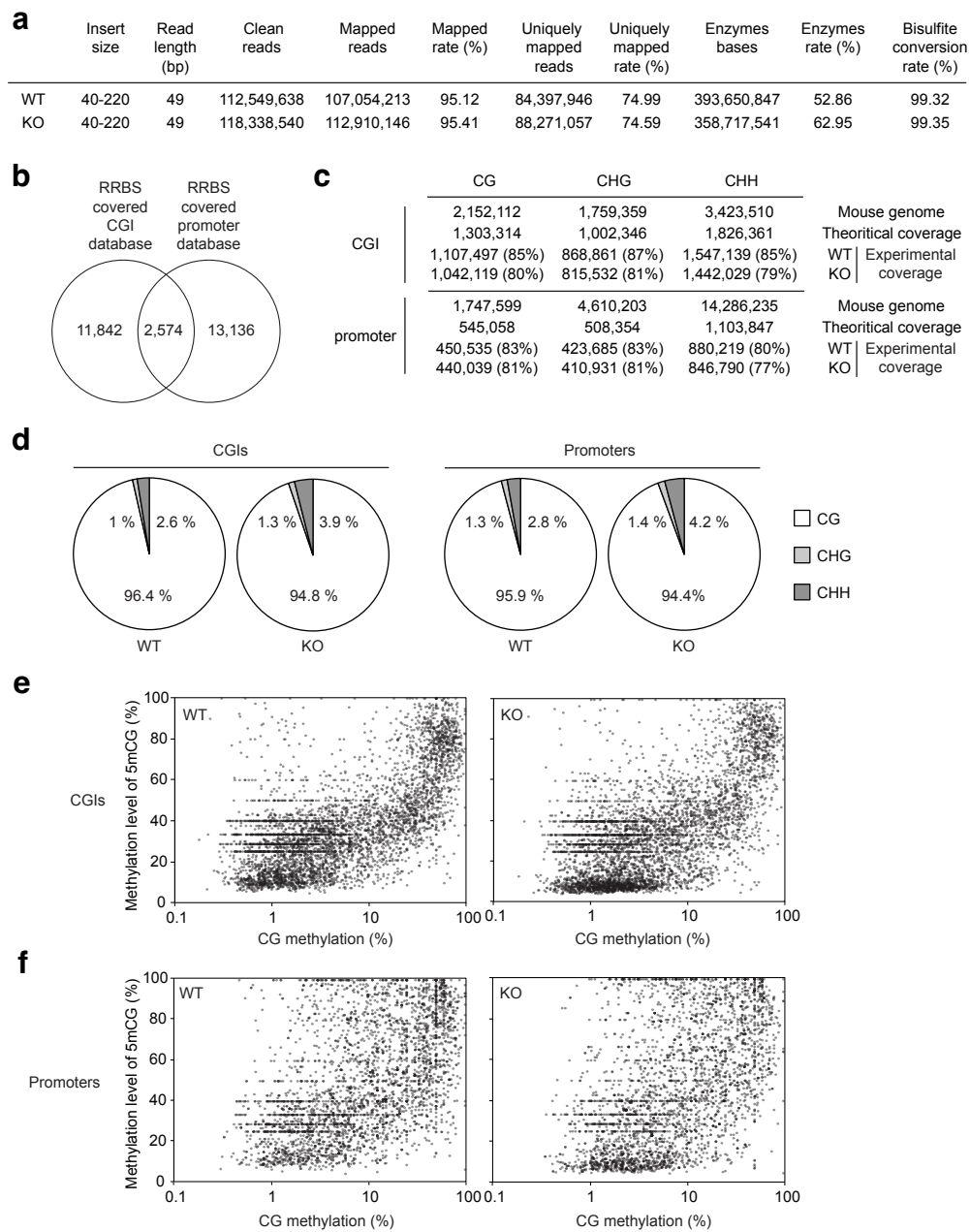

SupFig. 1

Supplement: Supplementary file 1 — Additional file 1: Supplementary Figure 1. Hypomethylation of promoters in the absence of MBD4 in MEFs. (a) Table summarizing the data obtained by RRBS after filtering and alignment of the raw reads. (b) Venn diagram showing the overlap between the two databases analyzed in this study. RRBS covered CGI and promoter databases correspond to databases described in methods but restricted to elements targeted by restriction enzyme digestion (14,416 and 15,710 elements, for CGIs and promoters respectively, which correspond to 90 % and 68 % of the corresponding mouse database). (c) Table listing the covered number of cytosines in each sequence context (CG, CHG and CHH, H represents non-G base). Theoretical values indicate cytosines located in theoretical enzyme cutting regions, and experimental values are the actual number of cytosines covered by sequencing reads. (d) Percentage of methylcytosines identified in CGIs or in promoters for WT and KO cells in each sequence context. (e-f) Dot blots representing the average methylation level of 5mCG as a function of the percentage of CG methylated per each CGI (d) or per each promoter (e) for WT (left panels) and KO (right panels) cells. [file 13046_2023_2882_MOESM1_ESM.pdf]

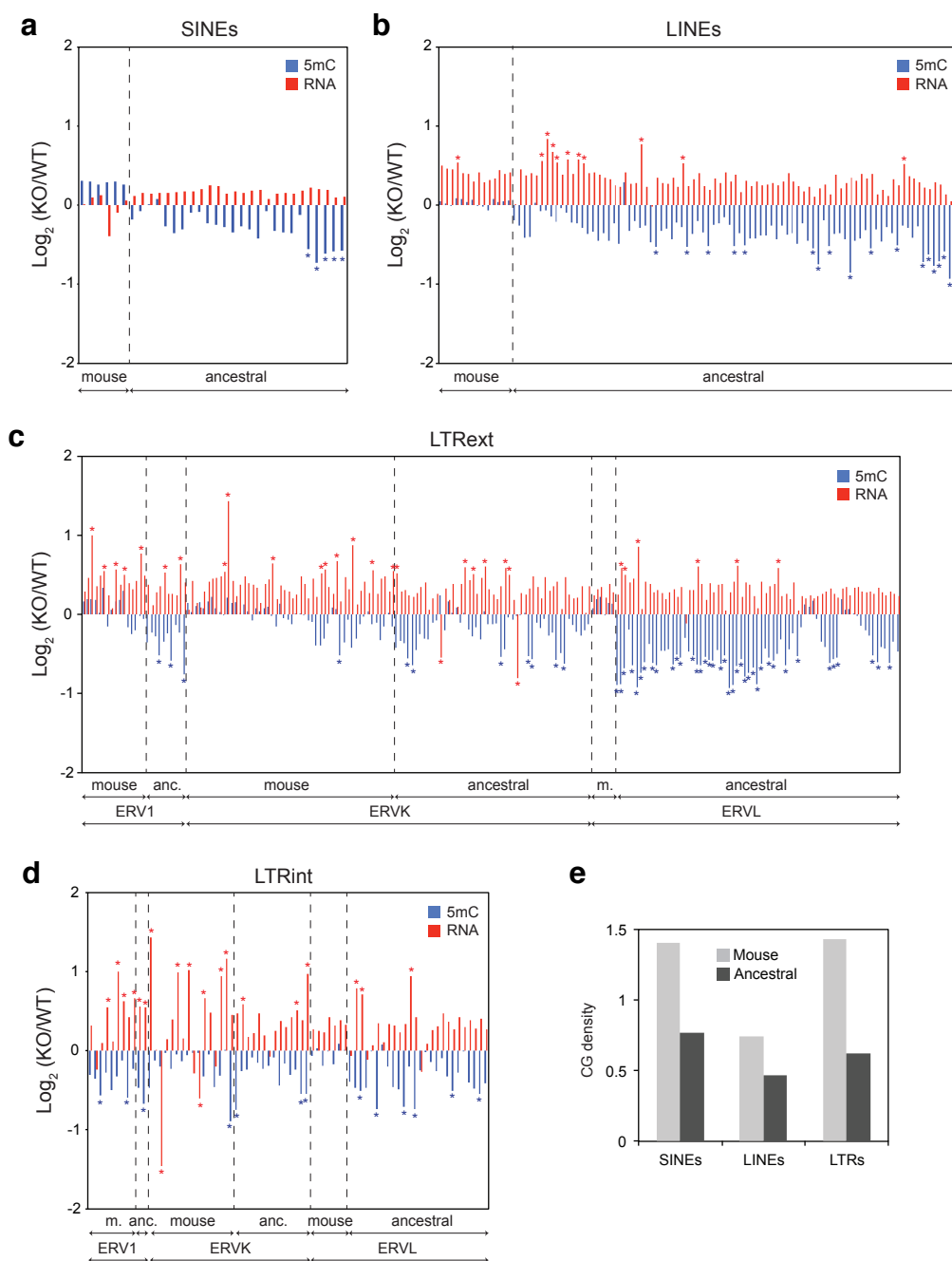

SupFig. 2

Supplement: Supplementary file 2 — Additional file 2: Supplementary Figure 2. Genome-wide demethylation and derepression of retrotransposons in Mbd4-/- MEFs. (a-d) Fold change (log2 ratio KO/WT) in 5mC enrichment or in the expression of SINE (a), LINE (b) and LTR (c-d) subfamilies in absence of MBD4 in MEFs. SINE and LINE subfamilies were arranged from the youngest to the oldest subfamilies to distinguish between lineage-specific (mouse) and ancestral families. Within the different LTR families, RMSK database distinguishes retro-elements corresponding to external domains (LTRext, containing the regulatory regions of the LTR) from those corresponding to internal domains (LTRint, containing the coding sequences of the proteins, necessary for the life cycle of the integrated viruses). Bearing this in mind, we carried out independent analyses for these two regions LTRext (c) and LTRint (d). LTR subfamilies were then sorted by classes (ERV1, ERVK and ERVL), and within each class of LTR, young mouse-specific LTR subfamilies were isolated from ancestral families. Asterisks (*) indicate significant difference (|log2 ratio fold change| > 0.5 and P < 0.05). (e) Average CG density (number of CG dinucleotides per 100 bp) of lineage-specific (mouse) and ancestral retro-elements in the mouse genome. [file 13046_2023_2882_MOESM2_ESM.pdf]

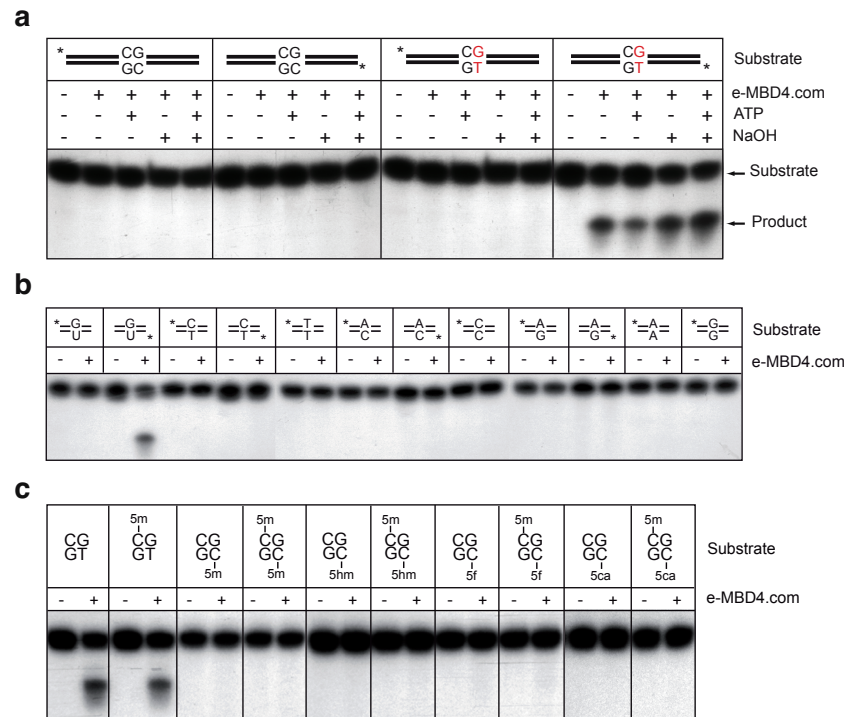

SupFig. 3

Supplement: Supplementary file 3 — Additional file 3: Supplementary Figure 3. The MBD4 complex exhibits G/T mismatch specific endonuclease activity. (a) In vitro glycosylase/nuclease assays. e-MBD4.com was mixed with the indicated substrates (* indicates the labeled strand), incubated for 20 minutes at 37°C and the products of the reaction were run on PAGE under denaturing conditions. Note that the generation of cut products does not require NaOH treatment. (b-c) e-MBD4.com were incubated with indicated substrates (* indicates the labeled strand) as described in (a). Reaction products were not treated with NaOH. [file 13046_2023_2882_MOESM3_ESM.pdf]
