# Supplementary material for: MBD4 loss results in global reactivation of promoters and retroelements with low methylated CpG density
Source: J Exp Clin Cancer Res. 2023 Nov 14;42:301. doi: 10.1186/s13046-023-02882-z (PMC10644448; doi:10.1186/s13046-023-02882-z)
Supplement: Supplementary file 10 — Additional file 10: Supplementary Table 10. Full list of peptides and proteins identified by mass spectrometry analysis of the epitope-tagged MBD4 complex purified from HeLa cells by double immunoaffinity. [file 13046_2023_2882_MOESM10_ESM.pdf]

GYGI LAB / TAPLIN BIOLOGICAL MASS SPEC FACILITY SearchWiz | Status | Browse

/home/www/production/html/saved_sets/17567.2.sumlist

668 peptides, **177** proteins Charge states: 1+ 2+ 3+ 4+ Sort: Reference desc html text dta Peptides Reload

17567.2 Lookup scanf scanl Rank Rank_Sp charge Ions premass _M_H_ mass_diff

peptide reference

Group 2/3+

Use best ion

R: H: Sequence:
R: H: Peptide type: tryptic
R: H: AA type: acidic
R: H: Mod AA type: acidic

XCorr 1+ 0
XCorr 2+ 0
XCorr 3+ 0
XCorr 4+ 0
dCn:
dCn2:

Rank (1 to n):
Low PPM1:
High PPM1:
Low CalPPM:
High CalPPM:
Vista Score:

Hide duplicate peptides

Protein matches				
**47**	**47**	3.502	0.00	UPSP:MBD4_HUMAN
**36**	**36**	3.529	0.00	UPSP:MLH1_HUMAN
**30**	**30**	3.218	0.00	UPSP:USP9X_HUMAN
**29**	**29**	3.512	0.00	UPSP:PMS2_HUMAN
**28**	**28**	3.438	0.00	UPSP:ANM5_HUMAN
**20**	**20**	4.164	0.00	UPTR:Q6LC01_HUMAN
**17**	**17**	3.352	0.00	UPSP:TBA2_HUMAN
**16**	**16**	3.480	0.00	UPSP:RUVB2_HUMAN
**16**	**16**	3.290	0.00	UPSP:KIF11_HUMAN
**12**	**12**	4.298	0.00	UPSP:NPM_HUMAN
**12**	**12**	3.485	0.00	UPSP:SIRT1_HUMAN
**10**	**10**	3.023	0.00	UPSP:VIME_HUMAN
9	9	3.559	0.00	UPSP:RUVB1_HUMAN
9	9	2.971	0.00	UPSP:DDB1_HUMAN
9	9	2.840	0.00	UPSP:PRDX1_HUMAN
8	8	3.680	0.00	UPSP:HSP7C_HUMAN
7	7	3.242	0.00	UPSP:K6PP_HUMAN
7	7	3.213	0.00	UPSP:GRP78_HUMAN
7	7	3.014	0.00	UPSP:PP2CG_HUMAN
7	7	2.912	0.00	UPSP:UBP7_HUMAN
7	7	2.883	0.00	UPSP:DNJA2_HUMAN
7	7	2.693	0.00	UPSP:HNRPM_HUMAN
6	6	4.844	0.00	UPSP:ICLN_HUMAN
6	6	3.793	0.00	UPSP:MEP50_HUMAN
6	6	3.253	0.00	UPSP:ANXA2_HUMAN
6	6	3.188	0.00	UPSP:TBB1_HUMAN
5	5	4.133	0.00	UPTR:Q8IWP6_HUMAN
5	5	4.093	0.00	UPSP:HNRPK_HUMAN
5	5	3.886	0.00	UPSP:RSSA_HUMAN
5	5	3.471	0.00	UPSP:TBB6_HUMAN
5	5	3.130	0.00	UPTR:Q5T8M8_HUMAN
5	5	3.127	0.00	UPTR:Q5VTE0_HUMAN
5	5	3.067	0.00	UPSP:DDX39_HUMAN
5	5	2.849	0.00	UPSP:MSH6_HUMAN
4	4	4.063	0.00	UPSP:DNJA1_HUMAN
4	4	3.747	0.00	UPSP:NDRG1_HUMAN
4	4	3.711	0.00	UPSP:RLA0_HUMAN
4	4	3.395	0.00	UPSP:RINI_HUMAN
4	4	3.390	0.00	UPTR:Q53HU2_HUMAN
4	4	3.144	0.00	UPTR:Q6FGB8_HUMAN
4	4	2.961	0.00	UPSP:HSP72_HUMAN
4	4	2.474	0.00	UPSP:HNRPU_HUMAN
4	4	2.412	0.00	UPSP:RS9_HUMAN
4	4	2.343	0.00	UPSP:MSH2_HUMAN
4	4	2.245	0.00	UPSP:MYPT1_HUMAN
3	3	3.953	0.00	UPSP:PMS1_HUMAN
3	3	3.748	0.00	UPTR:Q9NQS8_HUMAN
3	3	3.643	0.00	UPSP:RL7A_HUMAN
3	3	3.599	0.00	UPSP:HSP71_HUMAN
3	3	3.581	0.00	UPSP:2AAA_HUMAN
3	3	3.440	0.00	UPSP:2ABA_HUMAN
3	3	3.338	0.00	UPSP:MCM7_HUMAN
3	3	3.230	0.00	UPTR:Q59GP5_HUMAN
3	3	3.091	0.00	UPSP:TCPA_HUMAN
3	3	3.081	0.00	UPSP:SF3B3_HUMAN
3	3	3.049	0.00	UPSP:TCPG_HUMAN
3	3	2.958	0.00	UPTR:Q8WVX7_HUMAN
3	3	2.907	0.00	UPSP:RS6_HUMAN
3	3	2.855	0.00	UPSP:DDX48_HUMAN
3	3	2.819	0.00	UPTR:Q6IPX9_HUMAN
3	3	2.559	0.00	UPSP:FLNB_HUMAN
3	3	2.237	0.00	UPTR:Q6PJ43_HUMAN
2	2	5.828	0.00	UPTR:Q562X8_HUMAN
2	2	4.597	0.00	UPSP:HNRH1_HUMAN
2	2	4.467	0.00	UPSP:TCPE_HUMAN
2	2	4.428	0.00	UPTR:Q9BRB1_HUMAN
2	2	4.366	0.00	UPSP:TBA6_HUMAN
2	2	3.699	0.00	UPTR:Q53S41_HUMAN
2	2	3.663	0.00	UPSP:RS17_HUMAN
2	2	3.632	0.00	UPSP:TBB4Q_HUMAN
2	2	3.559	0.00	UPTR:Q53G25_HUMAN
2	2	3.506	0.00	GP:BC011752_1
2	2	3.498	0.00	UPSP:U2AF2_HUMAN
2	2	3.495	0.00	UPTR:Q9BYX7_HUMAN
2	2	3.462	0.00	UPSP:RS24_HUMAN
2	2	3.450	0.00	UPSP:RS23_HUMAN
2	2	3.449	0.00	UPSP:RL18_HUMAN
2	2	3.418	0.00	GP:AY891365_1
2	2	3.236	0.00	GP:AY892659_1
2	2	3.112	0.00	UPSP:COPA_HUMAN
2	2	3.089	0.00	UPSP:RL5_HUMAN
2	2	3.071	0.00	UPSP:PCBP2_HUMAN
2	2	3.041	0.00	UPSP:K6PL_HUMAN
2	2	3.041	0.00	UPSP:SAHH2_HUMAN
2	2	3.011	0.00	UPTR:Q9NXD9_HUMAN
2	2	2.982	0.00	UPSP:RS2_HUMAN
2	2	2.907	0.00	UPTR:Q5GGW2_HUMAN
2	2	2.868	0.00	UPSP:RL7_HUMAN
2	2	2.861	0.00	UPTR:Q498Y6_HUMAN
2	2	2.739	0.00	UPSP:RL26L_HUMAN
2	2	2.728	0.00	UPSP:RS4Y1_HUMAN
2	2	2.672	0.00	UPTR:Q9BS10_HUMAN
2	2	2.535	0.00	UPTR:Q6IPX4_HUMAN
2	2	2.510	0.00	GP:AY892156_1
2	2	2.497	0.00	UPSP:NOLA1_HUMAN
2	2	2.470	0.00	UPSP:H2A1A_HUMAN
2	2	2.451	0.00	UPTR:Q6ZSQ4_HUMAN
2	2	2.395	0.00	UPSP:PLEC1_HUMAN
2	2	2.373	0.00	UPSP:RS15A_HUMAN
2	2	2.368	0.00	UPSP:NOL1_HUMAN
2	2	2.351	0.00	UPSP:RL23_HUMAN
2	2	2.344	0.00	UPSP:TOP1_HUMAN
2	2	2.334	0.00	UPSP:NUCL_HUMAN
2	2	2.319	0.00	UPSP:RL27A_HUMAN
2	2	2.242	0.00	UPTR:Q6NXR8_HUMAN
2	2	2.083	0.00	UPSP:H2B1A_HUMAN
1	1	5.447	0.00	UPSP:DDX20_HUMAN
1	1	5.360	0.00	UPTR:Q53RR5_HUMAN
1	1	4.854	0.00	UPSP:XPO2_HUMAN
1	1	4.846	0.00	UPSP:TCPB_HUMAN
1	1	4.511	0.00	UPSP:TCPD_HUMAN
1	1	4.465	0.00	UPSP:DKC1_HUMAN
1	1	4.389	0.00	UPSP:RBBP7_HUMAN
1	1	4.332	0.00	UPTR:Q562P9_HUMAN
1	1	4.320	0.00	UPTR:Q5JP53_HUMAN
1	1	4.260	0.00	UPTR:Q4R5P9_MACFA
1	1	4.236	0.00	UPSP:IMDH2_HUMAN
1	1	4.152	0.00	UPTR:Q9NXF7_HUMAN
1	1	4.137	0.00	UPSP:H2B1B_HUMAN
1	1	4.114	0.00	UPTR:Q7Z5A3_HUMAN
1	1	4.077	0.00	UPSP:DHX15_HUMAN
1	1	3.981	0.00	UPTR:Q5VVC8_HUMAN
1	1	3.928	0.00	UPSP:RL10_HUMAN
1	1	3.845	0.00	UPSP:SART3_HUMAN
1	1	3.826	0.00	UPSP:RL38_HUMAN
1	1	3.799	0.00	UPTR:Q2NL65_HUMAN
1	1	3.797	0.00	UPTR:Q1JQ76_HUMAN
1	1	3.742	0.00	UPTR:Q6DHX9_HUMAN
1	1	3.742	0.00	UPSP:MCM3_HUMAN
1	1	3.735	0.00	UPTR:Q9UHX1_HUMAN
1	1	3.721	0.00	GP:AY891859_1
1	1	3.715	0.00	UPTR:Q6ICQ4_HUMAN
1	1	3.712	0.00	UPSP:HSP76_HUMAN
1	1	3.630	0.00	UPSP:RL29_HUMAN
1	1	3.581	0.00	UPSP:RIOK1_HUMAN
1	1	3.496	0.00	UPSP:HS70L_HUMAN
1	1	3.464	0.00	GP:AE006463_8
1	1	3.456	0.00	UPSP:AKP8L_HUMAN
1	1	3.444	0.00	UPSP:AHSA1_HUMAN
1	1	3.431	0.00	UPSP:SMD3_HUMAN
1	1	3.421	0.00	UPSP:K1967_HUMAN
1	1	3.413	0.00	GP:AY892286_1
1	1	3.408	0.00	UPSP:FXR1_HUMAN
1	1	3.378	0.00	UPSP:RL21_HUMAN
1	1	3.362	0.00	UPSP:RL3_HUMAN
1	1	3.355	0.00	UPTR:Q5TZB9_HUMAN
1	1	3.298	0.00	UPTR:Q53HV1_HUMAN
1	1	3.289	0.00	UPSP:KCTD5_HUMAN
1	1	3.285	0.00	UPSP:FLNA_HUMAN
1	1	3.207	0.00	UPSP:DDX17_HUMAN
1	1	3.180	0.00	UPSP:PRS4_HUMAN
1	1	3.161	0.00	UPSP:RL35_HUMAN
1	1	3.154	0.00	UPSP:LRP4_HUMAN
1	1	3.124	0.00	UPTR:Q6DRA9_HUMAN
1	1	3.111	0.00	UPSP:PAIRB_HUMAN
1	1	3.103	0.00	UPSP:IMB1_HUMAN
1	1	3.083	0.00	UPSP:DDX21_HUMAN
1	1	3.076	0.00	UPTR:Q5T8W0_HUMAN
1	1	3.037	0.00	UPTR:Q9H853_HUMAN
1	1	3.034	0.00	UPTR:Q6IRZ0_HUMAN
1	1	2.948	0.00	UPSP:PRS10_HUMAN
1	1	2.940	0.00	UPTR:Q4VXZ3_HUMAN
1	1	2.910	0.00	UPTR:Q5VVD0_HUMAN
1	1	2.839	0.00	UPTR:Q567Q7_HUMAN
1	1	2.816	0.00	GP:AY893966_1
1	1	2.805	0.00	UPTR:Q6S376_HUMAN
1	1	2.785	0.00	UPSP:MCM5_HUMAN
1	1	2.767	0.00	UPSP:LMNA_HUMAN
1	1	2.766	0.00	UPSP:EF2_HUMAN
1	1	2.751	0.00	UPTR:Q8TBW2_HUMAN
1	1	2.745	0.00	UPSP:CAYP2_HUMAN
1	1	2.616	0.00	UPSP:RL13_HUMAN
1	1	2.587	0.00	UPSP:RLA2_HUMAN
1	1	2.567	0.00	UPTR:Q5JVH5_HUMAN
1	1	2.557	0.00	UPSP:G6PD_HUMAN
1	1	2.552	0.00	UPTR:Q8WTY3_HUMAN
1	1	2.170	0.00	UPSP:KPYM_HUMAN

scanf	Rank	charge	Ions	dCn	dCn2	XCorr	Reference	Redu	Peptide
0427	1	2	18/20	0.1966	0.1966	3.4120	UPSP:MBD4_HUMAN	2	K.ADAESEPVAQK.S
2443	1	2	23/40	0.4830	0.4830	5.0527	UPSP:MBD4_HUMAN	2	K.DSEHNEKYEDTFLESEEIGTK.V
2186	1	2	25/30	0.5340	0.5340	4.2435	UPSP:MBD4_HUMAN	2	K.DVFM*PPSSSSELQESR.G
2497	1	2	24/30	0.4689	0.4689	4.1092	UPSP:MBD4_HUMAN	2	K.DVFMPPSSSSELQESR.G
1920	1	2	14/20	0.3646	0.3646	2.8646	UPSP:MBD4_HUMAN	2	K.EDEGVDDVNFR.K
1422	1	2	20/22	0.3545	0.3545	3.3337	UPSP:MBD4_HUMAN	2	K.EDEGVDDVNFRK.V
1706	1	2	14/16	0.2606	0.2606	2.4354	UPSP:MBD4_HUMAN	3	K.EDVAM*ELER.V
2018	1	2	13/16	0.2176	0.2176	2.5577	UPSP:MBD4_HUMAN	3	K.EDVAMELER.V
1118	1	2	12/16	0.3600	0.3600	2.2795	UPSP:MBD4_HUMAN	3	K.EHLHTDILK.R
0848	1	2	14/18	0.4461	0.4461	2.6667	UPSP:MBD4_HUMAN	3	K.EHLHTDILKR.G
1701	1	2	19/22	0.2445	0.2445	3.7514	UPSP:MBD4_HUMAN	4	K.FLEKYPSAEVAR.T
1775	1	2	14/14	0.3680	0.3680	2.8081	UPSP:MBD4_HUMAN	4	K.FSDEYLTK.Q
1946	1	2	25/32	0.5117	0.5117	4.5279	UPSP:MBD4_HUMAN	2	K.KDVFM*PPSSSSELQESR.G
2127	1	3	28/64	0.4258	0.4258	3.5890	UPSP:MBD4_HUMAN	2	K.KDVFMPPSSSSELQESR.G
4331	1	2	17/18	0.2245	0.2245	3.5188	UPSP:MBD4_HUMAN	4	K.LLIATIFLNR.T
2974	1	2	13/14	0.1004	0.1004	1.9293	UPSP:MBD4_HUMAN	4	K.M*AIPVLWK.F
3575	1	2	13/14	0.2995	0.2995	2.1881	UPSP:MBD4_HUMAN	4	K.MAIPVLWK.F
3631	1	3	34/68	0.2555	0.2555	3.9194	UPSP:MBD4_HUMAN	2	K.NGETSLKPEDFDFTVLSK.R
1680	1	2	15/16	0.3112	0.3112	2.5845	UPSP:MBD4_HUMAN	3	K.SSLANYLHK.N
3510	1	3	26/104	0.2463	0.2463	3.2191	UPSP:MBD4_HUMAN	2	K.SSLANYLHKNGETSLKPEDFDFTVLSK.R
3383	1	3	28/60	0.1420	0.1420	3.9794	UPSP:MBD4_HUMAN	3	K.TAGRFDVYFISPQGLK.F
0074	1	1	9/12	0.1009	0.1009	1.9446	UPSP:MBD4_HUMAN	3	K.TSGIINK.F
1667	1	2	11/14	0.1605	0.1605	1.9291	UPSP:MBD4_HUMAN	3	K.TSLYFSSK.Y
2671	1	2	23/26	0.4686	0.4686	5.6216	UPSP:MBD4_HUMAN	2	K.YEDTFLESEEIGTK.V
0515	1	2	10/12	0.1991	0.1991	2.2274	UPSP:MBD4_HUMAN	3	K.YGNDSYR.I
2191	1	2	17/20	0.4539	0.4539	4.1085	UPSP:MBD4_HUMAN	3	K.YHDWLWENHEK.L
2147	1	2	16/18	0.1690	0.1690	2.9778	UPSP:MBD4_HUMAN	3	K.YPIELHGIGK.Y
0857	1	2	12/14	0.2003	0.2003	2.2783	UPSP:MBD4_HUMAN	4	K.YPSAEVAR.T
4358	1	3	32/56	0.4493	0.4493	4.7415	UPSP:MBD4_HUMAN	4	R.DVSELLKPLGLYDLR.A
3864	1	2	19/22	0.4190	0.4190	3.6038	UPSP:MBD4_HUMAN	3	R.FDVYFISPQGLK.F
0596	1	1	11/20	0.3790	0.3790	2.5989	UPSP:MBD4_HUMAN	4	R.GAAPTVTSSER.L
2868	1	2	17/24	0.2666	0.2666	4.0962	UPSP:MBD4_HUMAN	3	R.GLSNFTSTHLLLK.E
3456	1	3	37/92	0.5568	0.5568	6.1761	UPSP:MBD4_HUMAN	2	R.GLSNFTSTHLLLKEDEGVDDVNFR.K
2920	1	3	32/96	0.4508	0.4508	5.7144	UPSP:MBD4_HUMAN	2	R.GLSNFTSTHLLLKEDEGVDDVNFRK.V
1286	1	2	17/18	0.2769	0.2769	3.3812	UPSP:MBD4_HUMAN	3	R.KEDVAM*ELER.V
1496	1	2	17/18	0.3690	0.3690	3.7072	UPSP:MBD4_HUMAN	3	R.KEDVAMELER.V
0758	1	2	16/18	0.3501	0.3501	3.3988	UPSP:MBD4_HUMAN	3	R.KEHLHTDILK.R
0687	1	2	18/20	0.3915	0.3915	3.5336	UPSP:MBD4_HUMAN	3	R.KEHLHTDILKR.G
1275	1	2	13/16	0.1220	0.1220	2.3496	UPSP:MBD4_HUMAN	3	R.KTSLYFSSK.Y
4458	1	2	24/30	0.4510	0.4510	5.4349	UPSP:MBD4_HUMAN	4	R.SPFNLVQETLFHDPWK.L
0715	1	2	17/20	0.3958	0.3958	3.3584	UPSP:MBD4_HUMAN	3	R.VGEDEEQM*M*IK.R
1434	1	2	15/20	0.0920	0.3413	3.4336	UPSP:MBD4_HUMAN	3	R.VGEDEEQM*MIK.R
1222	1	2	16/22	0.1014	0.2347	3.6306	UPSP:MBD4_HUMAN	3	R.VGEDEEQM*MIKR.S
1398	1	2	15/20	0.1149	0.2781	3.2862	UPSP:MBD4_HUMAN	3	R.VGEDEEQMM*IK.R
1033	1	2	18/22	0.0569	0.3156	4.1834	UPSP:MBD4_HUMAN	3	R.VGEDEEQMM*IKR.S
1919	1	2	15/20	0.3686	0.3686	3.6037	UPSP:MBD4_HUMAN	3	R.VGEDEEQMMIK.R
1526	1	2	19/22	0.3192	0.3192	4.2991	UPSP:MBD4_HUMAN	3	R.VGEDEEQMMIKR.S
3242	1	2	16/22	0.4354	0.4354	2.9409	UPSP:MLH1_HUMAN	7	K.AIETVYAAYLPK.N
0727	1	2	14/20	0.2145	0.2145	2.4459	UPSP:MLH1_HUMAN	6	K.ALKNPSEEYGK.I
4232	1	2	27/36	0.5059	0.5059	6.4185	UPSP:MLH1_HUMAN	6	K.HFETDGNILQLANLPDLYK.V
1551	1	2	11/12	0.0820	0.0820	2.4087	UPSP:MLH1_HUMAN	6	K.ILEVVGR.Y
0533	1	2	16/18	0.3146	0.3146	2.8076	UPSP:MLH1_HUMAN	6	K.KQGETVADVR.T
3417	1	3	38/120	0.5901	0.5901	5.9012	UPSP:MLH1_HUMAN	7	K.LDAFLQPLSKPLSSQPQAIVTEDKTDISSGR.A
2145	1	2	19/22	0.0546	0.0546	2.6927	UPSP:MLH1_HUMAN	5	K.LIQIQDNGTGIR.K
0254	1	2	11/14	0.2121	0.2121	2.2425	UPSP:MLH1_HUMAN	6	K.LLGSNSSR.M
4205	1	2	23/30	0.5691	0.5691	5.1160	UPSP:MLH1_HUMAN	5	K.LQSFEDLASISTYGFR.G
2270	1	2	12/14	0.0516	0.0516	2.1588	UPSP:MLH1_HUMAN	6	K.LYLLNTTK.L
2020	1	2	18/24	0.1378	0.1378	3.3075	UPSP:MLH1_HUMAN	7	K.MNGYISNANYSVK.K
0380	1	2	14/18	0.1495	0.1495	2.5113	UPSP:MLH1_HUMAN	7	K.NQSLEGDTTK.G
0722	1	2	14/16	0.1831	0.1831	2.5777	UPSP:MLH1_HUMAN	6	K.QGETVADVR.T
1857	1	2	14/16	0.1597	0.1597	2.7542	UPSP:MLH1_HUMAN	4	K.STSIQVIVK.E
0569	1	2	21/30	0.3753	0.3753	4.7782	UPSP:MLH1_HUMAN	6	K.STTSLTSSSTSGSSDK.V
1462	1	3	34/92	0.3260	0.3260	3.9586	UPSP:MLH1_HUMAN	6	K.STTSLTSSSTSGSSDKVYAHQMVR.T
0626	1	2	10/14	0.0726	0.0726	2.0885	UPSP:MLH1_HUMAN	7	K.VYAHQM*VR.T
2213	1	2	14/16	0.3334	0.3334	2.7390	UPSP:MLH1_HUMAN	6	K.WTVEHIVYK.A
2819	1	3	24/76	0.1279	0.1279	3.0664	UPSP:MLH1_HUMAN	7	R.ARQQDEEM*LELPAPAEVAAK.N
2496	1	2	22/34	0.5898	0.5898	4.8438	UPSP:MLH1_HUMAN	5	R.GEALASISHVAHVTITTK.T
1031	1	3	23/60	0.2440	0.2440	3.8896	UPSP:MLH1_HUMAN	6	R.HREDSDVEM*VEDDSRK.E
0997	1	3	26/60	0.1336	0.1336	3.3590	UPSP:MLH1_HUMAN	6	R.HREDSDVEMVEDDSRK.E
1875	1	2	16/28	0.3090	0.3090	3.7799	UPSP:MLH1_HUMAN	5	R.IAAGEVIQRPANAIK.E
4395	1	3	36/88	0.4802	0.4802	5.2251	UPSP:MLH1_HUMAN	7	R.IINLTSVLSLQEEINEQGHEVLR.E
2788	1	2	16/24	0.2756	0.2756	2.8375	UPSP:MLH1_HUMAN	7	R.KAIETVYAAYLPK.N
0921	1	2	13/14	0.0976	0.0976	2.1251	UPSP:MLH1_HUMAN	6	R.LDETVVNR.I
0974	1	2	13/14	0.1885	0.1885	2.4087	UPSP:MLH1_HUMAN	7	R.LVESTSLR.K
4064	1	2	23/38	0.4652	0.4652	5.1820	UPSP:MLH1_HUMAN	7	R.M*YFTQTLLPGLAGPSGEMVK.S
4252	1	2	24/38	0.4000	0.4000	4.3547	UPSP:MLH1_HUMAN	7	R.MYFTQTLLPGLAGPSGEMVK.S
2704	1	2	24/34	0.4081	0.4081	5.8703	UPSP:MLH1_HUMAN	7	R.QQDEEM*LELPAPAEVAAK.N
3181	1	2	25/34	0.5208	0.5208	5.8034	UPSP:MLH1_HUMAN	7	R.QQDEEMLELPAPAEVAAK.N
0822	1	2	16/16	0.2461	0.2461	3.5385	UPSP:MLH1_HUMAN	6	R.RLDETVVNR.I
0506	1	2	10/12	0.0923	0.0923	2.3736	UPSP:MLH1_HUMAN	6	R.SHILPPK.H
1973	1	2	14/16	0.1256	0.1256	1.8846	UPSP:MLH1_HUMAN	7	R.SIFGNAVSR.E
1997	1	2	17/22	0.3710	0.3710	2.7083	UPSP:MLH1_HUMAN	6	R.TLPNASTVDNIR.S
2290	1	2	19/24	0.4828	0.4828	3.9574	UPSP:MLH1_HUMAN	6	R.YSVHNAGISFSVK.K
4295	1	2	24/32	0.4833	0.4833	5.8138	UPSP:USP9X_HUMAN	3	K.ALTLQDLDNIWAAQAGK.H
1759	1	2	11/16	0.3087	0.3087	2.3640	UPSP:USP9X_HUMAN	3	K.EIYTNLGPR.L
2371	1	2	12/18	0.0599	0.0599	2.2086	UPSP:USP9X_HUMAN	3	K.ELLAFQTSEK.K
3364	1	2	15/16	0.4081	0.4081	3.1953	UPSP:USP9X_HUMAN	5	K.FNDYFEFPR.E
1650	1	2	10/12	0.0813	0.0813	1.8804	UPSP:USP9X_HUMAN	Â	K.HLSFVVR.F
2757	1	2	14/18	0.2949	0.2949	2.8069	UPSP:USP9X_HUMAN	Â	K.HSGDYFTLLR.H
2607	1	3	39/108	0.5294	0.5294	5.4280	UPSP:USP9X_HUMAN	2	K.LEGDNVNPESQLIQQSEQSESETAGSTK.Y
2272	1	2	13/14	0.0530	0.0530	2.7464	UPSP:USP9X_HUMAN	3	K.LIGQLNLK.D
2779	1	2	16/22	0.2731	0.2731	2.4000	UPSP:USP9X_HUMAN	3	K.LLLTAIGYGHVR.A
4495	1	2	21/40	0.5062	0.5062	3.9775	UPSP:USP9X_HUMAN	3	K.LSVPATFMLVSLDEGPGPPIK.Y
3390	1	2	16/18	0.1226	0.1226	2.8064	UPSP:USP9X_HUMAN	6	K.LYSVVSQLIR.C
1283	1	2	14/16	0.1469	0.1469	2.6843	UPSP:USP9X_HUMAN	Â	K.MNALNEVNK.V
0472	1	2	13/18	0.1188	0.1188	2.0557	UPSP:USP9X_HUMAN	Â	K.QDNESNVDPR.D
1472	1	2	14/18	0.2438	0.2438	2.6606	UPSP:USP9X_HUMAN	5	K.VLGGSFADQK.I
2617	1	2	13/16	0.3020	0.3020	2.5856	UPSP:USP9X_HUMAN	3	R.DGLTISFTK.I
2732	1	2	13/26	0.2111	0.2111	2.6861	UPSP:USP9X_HUMAN	2	R.ELDM*EPYTVAGVAK.L
2711	1	2	17/26	0.3427	0.3427	3.6402	UPSP:USP9X_HUMAN	2	R.ELDMEPYTVAGVAK.L
4026	1	2	12/26	0.3153	0.3153	2.4151	UPSP:USP9X_HUMAN	3	R.FWFAHNVLFNVSNR.F
2363	1	3	27/48	0.4531	0.4531	4.5113	UPSP:USP9X_HUMAN	1	R.HLQVIFGHLAASR.L
0922	1	2	19/20	0.1306	0.1306	4.3352	UPSP:USP9X_HUMAN	Â	R.LAQQISDEASR.Y
1914	1	2	11/12	0.1041	0.1041	2.0537	UPSP:USP9X_HUMAN	4	R.LQYYVPR.G
2763	1	3	29/88	0.3159	0.3159	3.6284	UPSP:USP9X_HUMAN	5	R.LVGVLVHSGQASGGHYYSYIIQR.N
2408	1	2	20/22	0.4470	0.4470	3.5424	UPSP:USP9X_HUMAN	2	R.MDTIDQDDELIR.Y
2347	1	2	15/22	0.2242	0.2242	2.8117	UPSP:USP9X_HUMAN	Â	R.NNFLPNADMETR.R
3299	1	3	28/72	0.3782	0.3782	4.3308	UPSP:USP9X_HUMAN	Â	R.TGETGIEETILEGHLGVTK.E
0565	1	2	15/16	0.3515	0.3515	3.0603	UPSP:USP9X_HUMAN	Â	R.VPGQEETVK.N
1407	1	2	19/22	0.3219	0.3219	3.8785	UPSP:USP9X_HUMAN	Â	R.VVIQSNDDIASR.A
3261	1	2	12/14	0.1148	0.1148	1.9794	UPSP:USP9X_HUMAN	3	R.WVVPVLPK.G
3189	1	3	33/84	0.5804	0.5804	5.2315	UPSP:USP9X_HUMAN	2	R.YISELAITTRPHQIIMPSAIER.S
0602	1	2	15/18	0.2251	0.2251	2.8255	UPSP:USP9X_HUMAN	3	R.YSHVQEVQER.L
0572	1	2	16/28	0.4972	0.4972	3.3441	UPSP:PMS2_HUMAN	6	K.EAVSSSHGPSDPTDR.A
0713	1	3	26/76	0.2661	0.2661	3.0678	UPSP:PMS2_HUMAN	6	K.EAVSSSHGPSDPTDRAEVEK.D
2801	1	2	19/32	0.2169	0.2169	4.5571	UPSP:PMS2_HUMAN	18	K.ELVENSLDAGATNIDLK.L
2207	1	2	12/18	0.2303	0.2303	2.0730	UPSP:PMS2_HUMAN	7	K.ENIGSVFGQK.Q
4275	1	2	24/30	0.5303	0.5303	5.7975	UPSP:PMS2_HUMAN	9	K.IQEFADLTQVETFGFR.G
2670	1	3	25/60	0.2342	0.2342	3.2485	UPSP:PMS2_HUMAN	7	K.LNEDIFIVDQHATDEK.Y
3832	1	2	20/32	0.5182	0.5182	4.5144	UPSP:PMS2_HUMAN	6	K.LNVSQQPLLDVEGNLIK.M
2267	1	2	24/32	0.4982	0.4982	4.6193	UPSP:PMS2_HUMAN	6	K.LVNTQDMSASQVDVAVK.I
0893	1	3	22/48	0.1084	0.1084	3.2632	UPSP:PMS2_HUMAN	7	K.M*HAADLEKPMVEK.Q
1420	1	3	22/48	0.2908	0.2908	3.4568	UPSP:PMS2_HUMAN	7	K.MHAADLEKPMVEK.Q
3652	1	2	18/30	0.3441	0.3441	3.5859	UPSP:PMS2_HUMAN	7	K.NGFDFVIDENAPVTER.A
0524	1	2	12/14	0.1297	0.1297	1.9842	UPSP:PMS2_HUMAN	7	K.QDQSPSLR.T
0516	1	3	24/60	0.2913	0.2913	4.2493	UPSP:PMS2_HUMAN	5	K.QLHHEAQQSEGEQNYR.K
2560	1	2	22/26	0.5312	0.5312	4.9658	UPSP:PMS2_HUMAN	7	K.SVMIGTALNTSEMK.K
2959	1	2	20/24	0.3196	0.3196	3.8233	UPSP:PMS2_HUMAN	6	K.TSLIGM*FDSDVNK.L
4497	1	3	32/116	0.5215	0.5215	5.0960	UPSP:PMS2_HUMAN	6	K.TSLIGM*FDSDVNKLNVSQQPLLDVEGNLIK.M
3623	1	2	20/24	0.4286	0.4286	4.5576	UPSP:PMS2_HUMAN	6	K.TSLIGMFDSDVNK.L
2877	1	2	15/24	0.1932	0.1932	2.2779	UPSP:PMS2_HUMAN	4	K.VVPLDFSM*SSLAK.R
3602	1	2	20/24	0.4330	0.4330	3.1248	UPSP:PMS2_HUMAN	4	K.VVPLDFSMSSLAK.R
2839	1	3	27/60	0.4736	0.4736	3.5344	UPSP:PMS2_HUMAN	7	K.YNFEMLQQHTVLQGQR.L
0908	1	2	19/26	0.3568	0.3568	3.1758	UPSP:PMS2_HUMAN	6	R.GM*LSSSTSGAISDK.G
1555	1	2	19/26	0.4350	0.4350	2.9327	UPSP:PMS2_HUMAN	6	R.GMLSSSTSGAISDK.G
4261	1	2	17/30	0.4172	0.4172	3.7420	UPSP:PMS2_HUMAN	8	R.GTTVSVQQLFSTLPVR.H
2012	1	2	14/20	0.1410	0.1410	2.6026	UPSP:PMS2_HUMAN	7	R.HIANLGVISQN.-
0983	1	2	10/14	0.2870	0.2870	2.1419	UPSP:PMS2_HUMAN	8	R.LMFDHNGK.I
1447	1	2	15/20	0.2199	0.2199	2.8996	UPSP:PMS2_HUMAN	6	R.LVNEVYHM*YNR.H
2021	1	2	15/20	0.1903	0.1903	3.1895	UPSP:PMS2_HUMAN	6	R.LVNEVYHMYNR.H
0488	1	3	23/44	0.3773	0.3773	3.8813	UPSP:PMS2_HUMAN	6	R.TGEEKKDVSISR.L
1956	1	2	15/26	0.3479	0.3479	2.1559	UPSP:PMS2_HUMAN	5	R.VLPQPTNLATPNTK.R
3841	1	3	25/48	0.3240	0.3240	4.4036	UPSP:ANM5_HUMAN	5	K.AAILPTSIFLTNK.K
3372	1	2	12/26	0.1341	0.1341	2.4661	UPSP:ANM5_HUMAN	5	K.AAILPTSIFLTNKK.G
4136	1	2	22/40	0.5765	0.5765	3.6542	UPSP:ANM5_HUMAN	4	K.DDGVSIPGEYTSFLAPISSSK.L
2365	1	2	18/28	0.2995	0.2995	2.8076	UPSP:ANM5_HUMAN	4	K.DRDPEAQFEM*PYVVR.L
2972	1	3	28/56	0.2739	0.2739	4.2157	UPSP:ANM5_HUMAN	4	K.DRDPEAQFEMPYVVR.L
3577	1	2	11/26	0.2139	0.2139	2.1249	UPSP:ANM5_HUMAN	5	K.DTNVQVLMVLGAGR.G
1541	1	2	12/14	0.1097	0.1097	2.8395	UPSP:ANM5_HUMAN	4	K.KGFPVLSK.M
4606	1	3	30/108	0.4245	0.4245	4.0437	UPSP:ANM5_HUMAN	5	K.NPNAVVTLENWQFEEWGSQVTVVSSDMR.E
0985	1	2	13/16	0.0857	0.0857	2.1422	UPSP:ANM5_HUMAN	3	K.REFIQEPAK.N
3173	1	3	37/76	0.3399	0.3399	4.6626	UPSP:ANM5_HUMAN	4	K.RIAVALEIGADLPSNHVIDR.W
3409	1	2	12/16	0.2319	0.2319	2.2625	UPSP:ANM5_HUMAN	4	K.TWM*WWHNFR.T
3626	1	2	13/16	0.2518	0.2518	2.7051	UPSP:ANM5_HUMAN	4	K.TWMWWHNFR.T
1509	1	2	16/18	0.4684	0.4684	3.8698	UPSP:ANM5_HUMAN	5	K.YSQYQQAIYK.C
1839	1	2	23/38	0.5564	0.5564	5.0715	UPSP:ANM5_HUMAN	4	R.DDIIENAPTTHTEEYSGEEK.T
0367	1	2	13/14	0.2321	0.2321	2.5850	UPSP:ANM5_HUMAN	4	R.DPM*IDNNR.Y
0680	1	2	12/14	0.1328	0.1328	2.6169	UPSP:ANM5_HUMAN	4	R.DPMIDNNR.Y
2963	1	2	14/16	0.1298	0.1298	2.3051	UPSP:ANM5_HUMAN	5	R.DWNTLIVGK.L
1343	1	1	11/14	0.1217	0.1217	2.3281	UPSP:ANM5_HUMAN	3	R.EFIQEPAK.N
2113	1	2	13/32	0.3259	0.3259	2.6977	UPSP:ANM5_HUMAN	4	R.EKDRDPEAQFEM*PYVVR.L
2666	1	3	30/64	0.3822	0.3822	4.5153	UPSP:ANM5_HUMAN	4	R.EKDRDPEAQFEMPYVVR.L
1751	1	2	15/16	0.2994	0.2994	2.7563	UPSP:ANM5_HUMAN	6	R.GPLVNASLR.A
3562	1	3	28/72	0.3312	0.3312	4.3743	UPSP:ANM5_HUMAN	4	R.IAVALEIGADLPSNHVIDR.W
1794	1	2	11/14	0.1706	0.1706	2.5322	UPSP:ANM5_HUMAN	4	R.SDLLLSGR.D
2067	1	3	26/68	0.3436	0.3436	3.3402	UPSP:ANM5_HUMAN	4	R.VLTNHIHTGHHSSMFWMR.V
2936	1	3	33/76	0.3310	0.3310	5.3855	UPSP:ANM5_HUMAN	5	R.VPEEEKDTNVQVLM*VLGAGR.G
3483	1	3	28/76	0.1440	0.1440	3.5538	UPSP:ANM5_HUMAN	5	R.VPEEEKDTNVQVLMVLGAGR.G
2424	1	2	14/18	0.2918	0.2918	3.2171	UPSP:ANM5_HUMAN	4	R.VPLVAPEDLR.D
3327	1	3	43/116	0.4546	0.4546	6.7788	UPSP:ANM5_HUMAN	4	R.VPLVAPEDLRDDIIENAPTTHTEEYSGEEK.T
1907	1	2	18/22	0.2344	0.2344	3.8407	UPTR:Q6LC01_HUMAN	19	K.EVDEQM*LNVQNK.N
1876	1	2	18/22	0.2467	0.2467	4.2173	UPTR:Q6LC01_HUMAN	19	K.EVDEQMLNVQNK.N
3359	1	3	40/104	0.3456	0.3456	6.2964	UPTR:Q6LC01_HUMAN	3	K.FWEVISDEHGIDPTGTYHGDSDLQLDR.I
4286	1	3	30/68	0.3409	0.3409	4.2865	UPTR:Q6LC01_HUMAN	19	K.GHYTEGAELVDSVLDVVR.K
4108	1	3	36/72	0.4243	0.4243	4.9109	UPTR:Q6LC01_HUMAN	19	K.GHYTEGAELVDSVLDVVRK.E
4322	1	2	25/32	0.4900	0.4900	5.5000	UPTR:Q6LC01_HUMAN	8	K.M*AVTFIGNSTAIQELFK.R
4525	1	2	16/32	0.3038	0.3038	3.5046	UPTR:Q6LC01_HUMAN	8	K.MAVTFIGNSTAIQELFK.R
4279	1	3	25/68	0.1268	0.1268	3.4163	UPTR:Q6LC01_HUMAN	8	K.MAVTFIGNSTAIQELFKR.I
2849	1	2	13/20	0.3522	0.3522	2.7841	UPTR:Q6LC01_HUMAN	24	K.RISEQFTAMFR.R
2882	1	2	18/28	0.3894	0.3894	3.7551	UPTR:Q6LC01_HUMAN	13	R.AILVDLEPGTM*DSVR.S
3350	1	2	23/28	0.2692	0.2692	4.6468	UPTR:Q6LC01_HUMAN	13	R.AILVDLEPGTMDSVR.S
2275	1	2	18/22	0.4335	0.4335	4.0569	UPTR:Q6LC01_HUMAN	17	R.IM*NTFSVVPSPK.V
2648	1	2	20/22	0.4439	0.4439	3.9462	UPTR:Q6LC01_HUMAN	17	R.IMNTFSVVPSPK.V
2463	1	2	16/18	0.4084	0.4084	3.4827	UPTR:Q6LC01_HUMAN	24	R.ISEQFTAM*FR.R
3215	1	2	17/18	0.4745	0.4745	3.7268	UPTR:Q6LC01_HUMAN	24	R.ISEQFTAMFR.R
1703	1	2	19/22	0.4464	0.4464	3.8705	UPTR:Q6LC01_HUMAN	3	R.ISVYYNEATGGK.Y
0990	1	3	27/60	0.2680	0.2680	4.2515	UPTR:Q6LC01_HUMAN	19	R.M*SM*KEVDEQM*LNVQNK.N
1593	1	3	26/60	0.1557	0.1557	3.6482	UPTR:Q6LC01_HUMAN	19	R.M*SM*KEVDEQMLNVQNK.N
4250	1	3	42/100	0.4182	0.4182	6.6101	UPTR:Q6LC01_HUMAN	14	R.SGPFGQIFRPDNFVFGQSGAGNNWAK.G
3315	1	2	15/16	0.2678	0.2678	2.5361	UPTR:Q6LC01_HUMAN	13	R.YLTVAAVFR.G
2082	1	2	17/18	0.3798	0.3798	3.2581	UPSP:TBA2_HUMAN	17	K.DVNAAIATIK.T
0358	1	2	15/16	0.3008	0.3008	2.7927	UPSP:TBA2_HUMAN	20	K.EDAANNYAR.G
2023	1	2	12/12	0.2077	0.2077	2.1678	UPSP:TBA2_HUMAN	22	K.FDLM*YAK.R
2468	1	2	12/12	0.1554	0.1554	2.1032	UPSP:TBA2_HUMAN	22	K.FDLMYAK.R
3477	1	2	23/38	0.5967	0.5967	5.6879	UPSP:TBA2_HUMAN	15	K.TIGGGDDSFNTFFSETGAGK.H
2855	1	2	22/34	0.4638	0.4638	4.2577	UPSP:TBA2_HUMAN	21	K.VGINYQPPTVVPGGDLAK.V
3130	1	3	28/76	0.3415	0.3415	3.9451	UPSP:TBA2_HUMAN	19	R.AFVHWYVGEGM*EEGEFSEAR.E
3423	1	3	27/76	0.3866	0.3866	3.7164	UPSP:TBA2_HUMAN	19	R.AFVHWYVGEGMEEGEFSEAR.E
4397	1	2	24/40	0.5365	0.5365	4.5345	UPSP:TBA2_HUMAN	18	R.FDGALNVDLTEFQTNLVPYPR.I
3404	1	3	29/60	0.4120	0.4120	3.7761	UPSP:TBA2_HUMAN	16	R.IHFPLATYAPVISAEK.A
2121	1	2	15/20	0.2915	0.2915	2.8287	UPSP:TBA2_HUMAN	20	R.LDHKFDLM*YAK.R
2136	1	3	23/40	0.2218	0.2218	3.2966	UPSP:TBA2_HUMAN	20	R.LDHKFDLMYAK.R
1094	1	2	10/12	0.1068	0.1068	1.8073	UPSP:TBA2_HUMAN	16	R.LSVDYGK.K
0750	1	2	13/14	0.2310	0.2310	2.5523	UPSP:TBA2_HUMAN	16	R.LSVDYGKK.S
2151	1	2	15/26	0.1017	0.1017	3.1232	UPSP:TBA2_HUMAN	19	R.NLDIERPTYTNLNR.L
2730	1	2	18/22	0.2900	0.2900	3.0366	UPSP:TBA2_HUMAN	19	R.QLFHPEQLITGK.E
2604	1	3	30/80	0.3435	0.3435	4.0978	UPSP:TBA2_HUMAN	18	R.QLFHPEQLITGKEDAANNYAR.G
2543	1	3	25/64	0.2861	0.2861	4.3505	UPSP:RUVB2_HUMAN	3	K.EVVHTSVLHEIDVINSR.T
1156	1	2	17/20	0.3245	0.3245	3.3461	UPSP:RUVB2_HUMAN	2	K.GTEVQVDDIKR.V
2022	1	2	13/16	0.2925	0.2925	2.5870	UPSP:RUVB2_HUMAN	4	K.TEALTQAFR.R
2274	1	2	18/24	0.5011	0.5011	3.8633	UPSP:RUVB2_HUMAN	4	K.TTEM*ETIYDLGTK.M
2923	1	2	19/24	0.4648	0.4648	4.5539	UPSP:RUVB2_HUMAN	4	K.TTEMETIYDLGTK.M
2950	1	2	15/18	0.2479	0.2479	2.6598	UPSP:RUVB2_HUMAN	4	R.AAGVVLEMIR.E
3789	1	2	20/30	0.4565	0.4565	4.2842	UPSP:RUVB2_HUMAN	3	R.ALESDMAPVLIMATNR.G
1399	1	2	16/22	0.2399	0.2399	2.6245	UPSP:RUVB2_HUMAN	4	R.AVLIAGQPGTGK.T
2595	1	2	15/20	0.3504	0.3504	2.5223	UPSP:RUVB2_HUMAN	4	R.GLGLDDALEPR.Q
2010	1	2	12/14	0.1988	0.1988	2.6098	UPSP:RUVB2_HUMAN	3	R.IGLETSLR.Y
0804	1	3	24/44	0.2488	0.2488	3.9833	UPSP:RUVB2_HUMAN	2	R.KGTEVQVDDIKR.V
2505	1	2	19/22	0.3075	0.3075	3.1200	UPSP:RUVB2_HUMAN	3	R.LLIVSTTPYSEK.D
0936	1	2	17/24	0.3733	0.3733	3.2416	UPSP:RUVB2_HUMAN	4	R.QASQGM*VGQLAAR.R
1659	1	2	19/24	0.3852	0.3852	3.6085	UPSP:RUVB2_HUMAN	4	R.QASQGMVGQLAAR.R
4298	1	2	25/30	0.4881	0.4881	5.2974	UPSP:RUVB2_HUMAN	3	R.TQGFLALFSGDTGEIK.S
3027	1	2	15/18	0.3375	0.3375	3.0320	UPSP:RUVB2_HUMAN	2	R.VYSLFLDESR.S
1818	1	3	26/56	0.2564	0.2564	3.1031	UPSP:KIF11_HUMAN	1	K.AVDQHNAEAQDIFGK.N
3209	1	3	35/72	0.3663	0.3663	5.3763	UPSP:KIF11_HUMAN	1	K.GLEEITVHNKDEVYQILEK.G
1607	1	2	17/18	0.3103	0.3103	3.5097	UPSP:KIF11_HUMAN	1	K.IGAVEEELNR.V
1754	1	2	15/20	0.0987	0.0987	2.2747	UPSP:KIF11_HUMAN	2	K.LDIPTGTTPQR.K
2892	1	2	13/14	0.1385	0.1385	1.9198	UPSP:KIF11_HUMAN	2	K.LMNLWTER.F
3326	1	2	21/26	0.2112	0.2112	3.9051	UPSP:KIF11_HUMAN	1	K.LNLVDLAGSENIGR.S
1660	1	2	14/20	0.3177	0.3177	2.9829	UPSP:KIF11_HUMAN	1	K.LTDNGTEFSVK.V
4181	1	2	20/26	0.3278	0.3278	5.0237	UPSP:KIF11_HUMAN	1	K.LTVQEEQIVELIEK.I
0634	1	2	9/12	0.0908	0.0908	1.8631	UPSP:KIF11_HUMAN	2	K.LVEESVK.H
0409	1	2	13/16	0.1885	0.1885	2.6184	UPSP:KIF11_HUMAN	1	K.TQELETTQK.H
2885	1	2	19/26	0.3953	0.3953	3.1624	UPSP:KIF11_HUMAN	1	R.EAGNINQSLLTLGR.V
1381	1	2	14/16	0.1591	0.1591	2.5878	UPSP:KIF11_HUMAN	1	R.ILQDSLGGR.T
1064	1	3	24/48	0.4182	0.4182	3.8694	UPSP:KIF11_HUMAN	2	R.SKVEETTEHLVTK.S
4363	1	2	24/36	0.5934	0.5934	5.2057	UPSP:KIF11_HUMAN	1	R.SPNEEYTWEEDPLAGIIPR.T
1449	1	2	12/14	0.2744	0.2744	2.6238	UPSP:KIF11_HUMAN	1	R.TLHQIFEK.L
1858	1	2	13/14	0.2490	0.2490	2.6169	UPSP:KIF11_HUMAN	1	R.VITALVER.T
0425	1	2	13/14	0.1775	0.1775	2.2843	UPSP:NPM_HUMAN	18	K.ADKDYHFK.V
3356	1	3	36/72	0.3069	0.3069	4.8403	UPSP:NPM_HUMAN	18	K.DELHIVEAEAM*NYEGSPIK.V
3570	1	2	27/36	0.4385	0.4385	4.8034	UPSP:NPM_HUMAN	18	K.DELHIVEAEAMNYEGSPIK.V
0830	1	2	14/16	0.3133	0.3133	2.7758	UPSP:NPM_HUMAN	18	K.GPSSVEDIK.A
4316	1	3	30/80	0.4062	0.4062	3.6871	UPSP:NPM_HUMAN	20	K.M*SVQPTVSLGGFEITPPVVLR.L
0589	1	1	10/12	0.1467	0.1467	1.9576	UPSP:NPM_HUMAN	19	K.MQASIEK.G
4377	1	2	24/40	0.5992	0.5992	5.4104	UPSP:NPM_HUMAN	20	K.MSVQPTVSLGGFEITPPVVLR.L
1298	1	2	18/24	0.3306	0.3306	4.3595	UPSP:NPM_HUMAN	18	K.VDNDENEHQLSLR.T
4127	1	3	32/52	0.3067	0.3067	4.5002	UPSP:NPM_HUMAN	11	R.M*TDQEAIQDLWQWR.K
4296	1	3	29/52	0.1647	0.1647	4.3452	UPSP:NPM_HUMAN	11	R.MTDQEAIQDLWQWR.K
3702	1	3	35/108	0.5692	0.5692	6.6702	UPSP:NPM_HUMAN	18	R.TVSLGAGAKDELHIVEAEAM*NYEGSPIK.V
3940	1	3	34/108	0.5081	0.5081	5.9417	UPSP:NPM_HUMAN	18	R.TVSLGAGAKDELHIVEAEAMNYEGSPIK.V
1389	1	2	15/18	0.2542	0.2542	2.6419	UPSP:SIRT1_HUMAN	1	K.FIALSDKEGK.L
0916	1	2	15/18	0.2772	0.2772	3.1134	UPSP:SIRT1_HUMAN	1	K.LSEITEKPPR.T
3796	1	3	31/84	0.3549	0.3549	4.9379	UPSP:SIRT1_HUMAN	1	K.VRPVALIPSSIPHEVPQILINR.E
2883	1	3	27/88	0.3068	0.3068	4.1766	UPSP:SIRT1_HUMAN	1	R.AGGAGFGTDGDDQEAINEAISVK.Q
0855	1	2	15/22	0.1132	0.1132	2.1677	UPSP:SIRT1_HUMAN	Â	R.EAASSPAGEPLR.K
2682	1	2	14/18	0.1126	0.1126	2.3012	UPSP:SIRT1_HUMAN	Â	R.GDIFNQVVPR.C
2942	1	2	14/28	0.4419	0.4419	3.3850	UPSP:SIRT1_HUMAN	1	R.NVESIAEQMENPDLK.N
3499	1	2	26/32	0.4948	0.4948	5.4316	UPSP:SIRT1_HUMAN	1	R.NYTQNIDTLEQVAGIQR.I
0263	1	2	14/14	0.1637	0.1637	2.5081	UPSP:SIRT1_HUMAN	Â	R.RDGPGLER.S
2780	1	3	25/52	0.1184	0.1184	3.1788	UPSP:SIRT1_HUMAN	1	R.RLDGNQYLFLPPNR.Y
0561	1	2	19/22	0.3827	0.3827	3.8059	UPSP:SIRT1_HUMAN	Â	R.SPGEPGGAAPER.E
4354	1	2	25/34	0.5259	0.5259	4.1726	UPSP:SIRT1_HUMAN	1	R.TSPPDSSVIVTLLDQAAK.S
1545	1	2	17/18	0.3665	0.3665	4.1011	UPSP:VIME_HUMAN	13	K.FADLSEAANR.N
3607	1	2	16/18	0.1468	0.1468	3.7816	UPSP:VIME_HUMAN	10	K.ILLAELEQLK.G
2013	1	2	14/20	0.1726	0.1726	2.6300	UPSP:VIME_HUMAN	11	R.EEAENTLQSFR.Q
1482	1	2	12/12	0.0808	0.0808	2.1292	UPSP:VIME_HUMAN	11	R.FANYIDK.V
4362	1	2	22/26	0.2590	0.2590	2.9830	UPSP:VIME_HUMAN	11	R.ISLPLPNFSSLNLR.E
2843	1	3	22/52	0.1827	0.1827	3.5337	UPSP:VIME_HUMAN	12	R.KVESLQEEIAFLKK.L
0768	1	2	15/18	0.2862	0.2862	2.7369	UPSP:VIME_HUMAN	13	R.QDVDNASLAR.L
0645	1	2	12/16	0.2172	0.2172	2.5329	UPSP:VIME_HUMAN	10	R.QVDQLTNDK.A
1931	1	2	21/26	0.4025	0.4025	3.9429	UPSP:VIME_HUMAN	10	R.SLYASSPGGVYATR.S
0648	1	2	10/14	0.0875	0.0875	1.8614	UPSP:VIME_HUMAN	10	R.SYVTTSTR.T
2085	1	2	16/20	0.2593	0.2593	2.9263	UPSP:RUVB1_HUMAN	5	K.GLGLDESGLAK.Q
0111	1	2	12/14	0.0514	0.0514	2.6705	UPSP:RUVB1_HUMAN	4	K.ILADQQDK.Y
3307	1	2	18/20	0.2780	0.2780	3.3956	UPSP:RUVB1_HUMAN	5	K.LDPSIFESLQK.E
0867	1	2	21/24	0.4326	0.4326	4.0767	UPSP:RUVB1_HUMAN	6	K.QAASGLVGQENAR.E
4080	1	3	31/52	0.4130	0.4130	4.5412	UPSP:RUVB1_HUMAN	5	K.TALALAIAQELGSK.V
4162	1	2	23/30	0.4272	0.4272	4.2562	UPSP:RUVB1_HUMAN	5	R.ALESSIAPIVIFASNR.G
1877	1	2	15/22	0.2571	0.2571	2.7055	UPSP:RUVB1_HUMAN	5	R.AVLLAGPPGTGK.T
3451	1	3	29/68	0.2637	0.2637	3.3541	UPSP:RUVB1_HUMAN	4	R.GTEDITSPHGIPLDLLDR.V
4053	1	2	15/26	0.3966	0.3966	4.1062	UPSP:RUVB1_HUMAN	4	R.YSVQLLTPANLLAK.I
3593	1	3	24/92	0.3370	0.3370	3.9154	UPSP:DDB1_HUMAN	2	K.LGKDPNTYFIVGTAMVYPEEAEPK.Q
1958	1	2	15/18	0.2657	0.2657	2.7118	UPSP:DDB1_HUMAN	2	K.LLASINSTVR.L
2568	1	2	13/16	0.1509	0.1509	2.4114	UPSP:DDB1_HUMAN	2	K.LVFSNVNLK.E
2074	1	2	17/20	0.2888	0.2888	3.1005	UPSP:DDB1_HUMAN	2	K.VTLGTQPTVLR.T
0879	1	2	11/12	0.1046	0.1046	1.9108	UPSP:DDB1_HUMAN	6	K.VVEELTR.I
3006	1	2	15/24	0.2640	0.2640	3.1565	UPSP:DDB1_HUMAN	2	R.ALYYLQIHPQELR.Q
2352	1	2	15/18	0.3122	0.3122	2.4990	UPSP:DDB1_HUMAN	2	R.IVVFQYSDGK.L
1074	1	2	12/14	0.1192	0.1192	2.1806	UPSP:DDB1_HUMAN	2	R.LGDSQLVK.L
3929	1	3	36/72	0.4645	0.4645	4.8567	UPSP:DDB1_HUMAN	2	R.SVLLLAYKPMEGNFEEIAR.D
1553	1	2	12/14	0.2981	0.2981	2.6163	UPSP:PRDX1_HUMAN	4	K.ADEGISFR.G
1192	1	2	14/20	0.2716	0.2716	2.5044	UPSP:PRDX1_HUMAN	3	K.ATAVM*PDGQFK.D
1770	1	2	14/20	0.1795	0.1795	2.4733	UPSP:PRDX1_HUMAN	3	K.ATAVMPDGQFK.D
0825	1	2	14/16	0.1326	0.1326	2.4941	UPSP:PRDX1_HUMAN	3	K.IGHPAPNFK.A
3411	1	2	20/30	0.3330	0.3330	3.8490	UPSP:PRDX1_HUMAN	3	K.QGGLGPMNIPLVSDPK.R
2901	1	2	11/14	0.2093	0.2093	2.6418	UPSP:PRDX1_HUMAN	7	R.GLFIIDDK.G
2577	1	2	16/18	0.3724	0.3724	3.3803	UPSP:PRDX1_HUMAN	4	R.LVQAFQFTDK.H
2369	1	2	16/20	0.3469	0.3469	3.0014	UPSP:PRDX1_HUMAN	9	R.QITVNDLPVGR.S
2137	1	2	16/18	0.2836	0.2836	2.5985	UPSP:PRDX1_HUMAN	3	R.TIAQDYGVLK.A
2969	1	2	20/22	0.2380	0.2380	3.7360	UPSP:HSP7C_HUMAN	4	K.DAGTIAGLNVLR.I
2310	1	3	27/64	0.2422	0.2422	4.0936	UPSP:HSP7C_HUMAN	4	K.LDKSQIHDIVLVGGSTR.I
0563	1	3	26/52	0.2447	0.2447	3.0625	UPSP:HSP7C_HUMAN	6	K.NQTAEKEEFEHQQK.E
2405	1	2	20/28	0.4025	0.4025	3.6723	UPSP:HSP7C_HUMAN	3	K.NQVAMNPTNTVFDAK.R
2805	1	2	18/20	0.3237	0.3237	4.0115	UPSP:HSP7C_HUMAN	6	K.NSLESYAFNMK.A
3506	1	2	22/26	0.4617	0.4617	4.1631	UPSP:HSP7C_HUMAN	4	K.SFYPEEVSSMVLTK.M
3072	1	3	28/68	0.4074	0.4074	4.1545	UPSP:HSP7C_HUMAN	4	K.TVTNAVVTVPAYFNDSQR.Q
0681	1	2	13/14	0.1145	0.1145	2.5477	UPSP:HSP7C_HUMAN	4	R.LSKEDIER.M
4014	1	2	26/40	0.6017	0.6017	4.7368	UPSP:K6PP_HUMAN	5	K.EIGWTDVGGWTGQGGSILGTK.R
4157	1	2	15/20	0.2915	0.2915	2.4997	UPSP:K6PP_HUMAN	4	K.EWSGLLEELAR.N
1431	1	2	12/14	0.1580	0.1580	2.4233	UPSP:K6PP_HUMAN	5	K.LPDDQIPK.T
2281	1	2	16/22	0.4121	0.4121	3.8678	UPSP:K6PP_HUMAN	6	R.DLQSNVEHLTEK.M
3722	1	3	23/64	0.2351	0.2351	3.1628	UPSP:K6PP_HUMAN	3	R.IIEVVDAIMTTAQSHQR.T
3912	1	2	22/24	0.3989	0.3989	3.8227	UPSP:K6PP_HUMAN	6	R.MLAIYDGFDGFAK.G
0169	1	2	11/14	0.2733	0.2733	2.1804	UPSP:K6PP_HUMAN	5	R.VGIADGHR.M
3022	1	2	14/22	0.0971	0.0971	2.3110	UPSP:GRP78_HUMAN	1	K.ELEEIVQPIISK.L
0761	1	2	12/16	0.0972	0.0972	2.0392	UPSP:GRP78_HUMAN	1	K.ITITNDQNR.L
2277	1	2	21/28	0.4034	0.4034	4.6607	UPSP:GRP78_HUMAN	1	K.NQLTSNPENTVFDAK.R
3942	1	2	17/26	0.3659	0.3659	3.0533	UPSP:GRP78_HUMAN	1	K.TFAPEEISAMVLTK.M
3230	1	2	21/30	0.2358	0.2358	3.8400	UPSP:GRP78_HUMAN	18	R.IINEPTAAAIAYGLDK.R
2581	1	2	16/26	0.4562	0.4562	2.8733	UPSP:GRP78_HUMAN	1	R.ITPSYVAFTPEGER.L
1191	1	2	19/20	0.2820	0.2820	3.7122	UPSP:GRP78_HUMAN	26	R.VEIIANDQGNR.I
3432	1	2	18/22	0.4404	0.4404	3.1836	UPSP:PP2CG_HUMAN	2	K.ALEDAFLAIDAK.L
1014	1	2	17/20	0.2157	0.2157	2.6990	UPSP:PP2CG_HUMAN	2	K.AYTGFSSNSER.G
2990	1	2	22/32	0.3862	0.3862	3.4433	UPSP:PP2CG_HUMAN	4	K.EEPGSDSGTTAVVALIR.G
1131	1	3	24/68	0.2167	0.2167	3.4331	UPSP:PP2CG_HUMAN	2	K.ELAQIAGRPTEDEDEKEK.V
3762	1	2	19/30	0.0974	0.0974	2.5379	UPSP:PP2CG_HUMAN	4	K.NLPPEEQMISALPDIK.V
1376	1	2	11/14	0.1039	0.1039	2.0794	UPSP:PP2CG_HUMAN	4	R.AIGDHFYK.R
4197	1	2	19/26	0.4747	0.4747	3.7241	UPSP:PP2CG_HUMAN	1	R.LPLPYGFSAMQGWR.V
3468	1	3	31/76	0.3939	0.3939	4.1712	UPSP:UBP7_HUMAN	1	K.LSEVLQAVTDHDIPQQLVER.L
3041	1	2	15/28	0.2455	0.2455	2.2230	UPSP:UBP7_HUMAN	1	K.TIPNDPGFVVTLSNR.M
4337	1	3	30/84	0.3425	0.3425	3.6716	UPSP:UBP7_HUMAN	1	R.AGFIQDTSLILYEEVKPNLTER.I
2589	1	2	12/18	0.2852	0.2852	2.0892	UPSP:UBP7_HUMAN	1	R.IQSLLDIQEK.E
2546	1	2	12/14	0.2336	0.2336	2.7984	UPSP:UBP7_HUMAN	1	R.LLEIVSYK.I
4404	1	2	13/22	0.1576	0.1576	2.7949	UPSP:UBP7_HUMAN	1	R.LNTDPMLLQFFK.S
1547	1	2	13/16	0.2219	0.2219	2.6329	UPSP:UBP7_HUMAN	1	R.VLLDNVENK.M
0617	1	2	10/12	0.1682	0.1682	1.8401	UPSP:DNJA2_HUMAN	1	K.EHEVFQR.D
3795	1	2	18/28	0.3535	0.3535	3.6878	UPSP:DNJA2_HUMAN	2	K.FDVQFPENNWINPDK.L
1361	1	2	13/14	0.2064	0.2064	2.9926	UPSP:DNJA2_HUMAN	2	K.ILEVHVDK.G
2939	1	3	24/68	0.3079	0.3079	3.0149	UPSP:DNJA2_HUMAN	Â	K.LYDILGVPPGASENELKK.A
2668	1	2	16/18	0.3203	0.3203	3.3888	UPSP:DNJA2_HUMAN	2	K.VSLEDLYNGK.T
1302	1	2	14/18	0.3876	0.3876	3.0533	UPSP:DNJA2_HUMAN	2	R.DGNDLHMTYK.I
0700	1	2	11/12	0.1745	0.1745	2.2060	UPSP:DNJA2_HUMAN	1	R.YGEQGLR.E
4028	1	2	16/30	0.3833	0.3833	3.0586	UPSP:HNRPM_HUMAN	6	K.VGEVTYVELLM*DAEGK.S
4281	1	2	17/30	0.3297	0.3297	3.3965	UPSP:HNRPM_HUMAN	6	K.VGEVTYVELLMDAEGK.S
0763	1	2	15/18	0.2758	0.2758	2.5296	UPSP:HNRPM_HUMAN	8	R.ADILEDKDGK.S
3584	1	2	14/20	0.3338	0.3338	2.8847	UPSP:HNRPM_HUMAN	6	R.AFITNIPFDVK.W
3051	1	2	18/24	0.3910	0.3910	2.9827	UPSP:HNRPM_HUMAN	8	R.LGSTVFVANLDYK.V
0213	1	2	11/14	0.1066	0.1066	2.1168	UPSP:HNRPM_HUMAN	21	R.M*GANSLER.M
0412	1	2	10/12	0.0997	0.0997	1.8836	UPSP:HNRPM_HUMAN	10	R.VGQTIER.M
2731	1	3	41/108	0.4605	0.4605	5.6062	UPSP:ICLN_HUMAN	3	K.FEEESKEPVADEEEEDSDDDVEPITEFR.F
2571	1	2	17/24	0.1005	0.1005	3.6749	UPSP:ICLN_HUMAN	3	K.GLGTGTLYIAESR.L
2861	1	2	22/36	0.5132	0.5132	4.2376	UPSP:ICLN_HUMAN	4	R.LEGMLSQSVSSQYNMAGVR.T
1510	1	2	15/22	0.2092	0.2092	2.1438	UPSP:ICLN_HUMAN	2	R.QQPDTEAVLNGK.G
2812	1	3	47/120	0.5401	0.5401	6.7426	UPSP:ICLN_HUMAN	3	R.TEDSIRDYEDGM*EVDTTPTVAGQFEDADVDH.-
3225	1	3	47/120	0.5814	0.5814	6.6576	UPSP:ICLN_HUMAN	3	R.TEDSIRDYEDGMEVDTTPTVAGQFEDADVDH.-
3966	1	2	21/26	0.4527	0.4527	3.9944	UPSP:MEP50_HUMAN	3	K.VWDLAQQVVLSSYR.A
3770	1	3	30/92	0.3768	0.3768	3.5843	UPSP:MEP50_HUMAN	3	K.YEHDDIVSTVSVLSSGTQAVSGSK.D
4741	1	3	28/100	0.4492	0.4492	4.4103	UPSP:MEP50_HUMAN	3	R.GILVASDSGAVELWELDENETLIVSK.F
1544	1	2	15/24	0.3017	0.3017	2.9659	UPSP:MEP50_HUMAN	2	R.KETPPPLVPPAAR.E
2864	1	2	22/28	0.4913	0.4913	4.7158	UPSP:MEP50_HUMAN	2	R.SDGALLLGASSLSGR.C
2672	1	3	29/64	0.3003	0.3003	3.0901	UPSP:MEP50_HUMAN	2	R.YRSDGALLLGASSLSGR.C
4209	1	2	14/26	0.2961	0.2961	3.0495	UPSP:ANXA2_HUMAN	7	K.GVDEVTIVNILTNR.S
2660	1	2	16/20	0.3882	0.3882	3.3848	UPSP:ANXA2_HUMAN	8	K.SLYYYIQQDTK.G
2689	1	3	25/64	0.3791	0.3791	3.8425	UPSP:ANXA2_HUMAN	7	K.TDLEKDIISDTSGDFRK.L
1401	1	2	18/20	0.4574	0.4574	3.8902	UPSP:ANXA2_HUMAN	8	K.TPAQYDASELK.A
2553	1	2	14/18	0.0720	0.0720	2.0226	UPSP:ANXA2_HUMAN	8	R.DALNIETAIK.T
1318	1	2	16/18	0.2952	0.2952	3.3276	UPSP:ANXA2_HUMAN	8	R.TNQELQEINR.V
2640	1	2	16/18	0.4133	0.4133	3.4882	UPSP:TBB1_HUMAN	36	K.LAVNM*VPFPR.L
3188	1	2	17/18	0.3682	0.3682	3.7932	UPSP:TBB1_HUMAN	36	K.LAVNMVPFPR.L
2261	1	2	16/18	0.2332	0.2332	3.0236	UPSP:TBB1_HUMAN	36	R.FPGQLNADLR.K
1950	1	3	18/40	0.2019	0.2019	3.0234	UPSP:TBB1_HUMAN	36	R.FPGQLNADLRK.L
2637	1	2	13/22	0.1688	0.1688	2.3813	UPSP:TBB1_HUMAN	Â	R.IM*NSFSVMPSPK.V
2778	1	2	17/20	0.3055	0.3055	3.4172	UPSP:TBB1_HUMAN	36	R.KLAVNMVPFPR.L
3363	1	3	37/104	0.5197	0.5197	5.5800	UPTR:Q8IWP6_HUMAN	4	K.FWEVISDEHGIDPTGTYHGDSDLQLER.I
4361	1	2	15/32	0.2532	0.2532	3.3626	UPTR:Q8IWP6_HUMAN	Â	K.MPATFIGNSTAIQELFK.R
3053	1	2	22/28	0.2472	0.2472	3.6794	UPTR:Q8IWP6_HUMAN	10	R.ALTVPELTQQM*FDAK.N
4039	1	2	20/28	0.4059	0.4059	3.4374	UPTR:Q8IWP6_HUMAN	10	R.ALTVPELTQQMFDAK.N
1750	1	2	20/22	0.3721	0.3721	4.6055	UPTR:Q8IWP6_HUMAN	2	R.INVYYNEATGGK.Y
4036	1	2	16/22	0.3918	0.3918	3.1739	UPSP:HNRPK_HUMAN	7	K.IILDLISESPIK.G
1312	1	3	29/52	0.3455	0.3455	4.3494	UPSP:HNRPK_HUMAN	6	K.RPAEDMEEEQAFKR.S
3370	1	2	22/36	0.4082	0.4082	5.2728	UPSP:HNRPK_HUMAN	6	R.GSYGDLGGPIITTQVTIPK.D
4128	1	3	29/88	0.3467	0.3467	3.6420	UPSP:HNRPK_HUMAN	5	R.IITITGTQDQIQNAQYLLQNSVK.Q
2904	1	3	29/56	0.3421	0.3421	4.0249	UPSP:HNRPK_HUMAN	7	R.LLIHQSLAGGIIGVK.G
1520	1	2	22/24	0.4118	0.4118	4.1470	UPSP:RSSA_HUMAN	10	K.FAAATGATPIAGR.F
3886	1	3	32/88	0.3304	0.3304	4.4990	UPSP:RSSA_HUMAN	9	K.FLAAGTHLGGTNLDFQMEQYIYK.R
3090	1	2	23/32	0.5490	0.5490	4.9617	UPSP:RSSA_HUMAN	9	R.AIVAIENPADVSVISSR.N
3890	1	2	18/28	0.3314	0.3314	3.6440	UPSP:RSSA_HUMAN	8	R.FTPGTFTNQIQAAFR.E
1851	1	2	11/14	0.2297	0.2297	2.1795	UPSP:RSSA_HUMAN	9	R.LLVVTDPR.A
4478	1	3	31/96	0.2898	0.2898	3.5418	UPSP:TBB6_HUMAN	Â	K.LTTPTYGDLNHLVSATMSGVTTSLR.F
3725	1	3	25/52	0.3033	0.3033	3.7945	UPSP:TBB6_HUMAN	25	K.NSSYFVEWIPNNVK.V
3217	1	2	14/18	0.1493	0.1493	3.2962	UPSP:TBB6_HUMAN	Â	R.ISEQFSAM*FR.R
3415	1	3	30/52	0.2336	0.2336	3.4044	UPSP:TBB6_HUMAN	24	R.LHFFM*PGFAPLTSR.G
4116	1	3	29/52	0.2620	0.2620	3.3160	UPSP:TBB6_HUMAN	24	R.LHFFMPGFAPLTSR.G
0840	1	2	16/18	0.2677	0.2677	2.7421	UPTR:Q5T8M8_HUMAN	20	K.AGFAGDDAPR.A
0698	1	2	18/20	0.3715	0.3715	3.4316	UPTR:Q5T8M8_HUMAN	20	K.DSYVGDEAQSK.R
0642	1	2	19/22	0.3842	0.3842	3.1449	UPTR:Q5T8M8_HUMAN	20	K.DSYVGDEAQSKR.G
2921	1	2	19/30	0.3951	0.3951	4.4672	UPTR:Q5T8M8_HUMAN	10	K.YPIEHGIITNWDDMEK.I
0888	1	2	12/20	0.1312	0.1312	1.8664	UPTR:Q5T8M8_HUMAN	21	R.HQGVMVGMGQK.D
0897	1	2	10/18	0.2979	0.2979	2.0974	UPTR:Q5VTE0_HUMAN	19	K.STTTGHLIYK.C
2138	1	3	28/56	0.3977	0.3977	3.9082	UPTR:Q5VTE0_HUMAN	19	K.THINIVVIGHVDSGK.S
2361	1	3	21/44	0.2498	0.2498	3.5168	UPTR:Q5VTE0_HUMAN	16	K.YYVTIIDAPGHR.D
3622	1	3	26/92	0.3145	0.3145	4.0341	UPTR:Q5VTE0_HUMAN	22	R.VETGVLKPGMVVTFAPVNVTTEVK.S
0876	1	2	9/10	0.1354	0.1354	2.0786	UPTR:Q5VTE0_HUMAN	20	R.YEEIVK.E
2814	1	2	20/26	0.4792	0.4792	3.8593	UPSP:DDX39_HUMAN	10	K.GLAITFVSDENDAK.I
2641	1	3	19/44	0.2802	0.2802	3.3432	UPSP:DDX39_HUMAN	11	K.LTLHGLQQYYVK.L
3802	1	2	15/20	0.3289	0.3289	2.9352	UPSP:DDX39_HUMAN	4	K.VSVFFGGLSIK.K
2205	1	2	11/12	0.0860	0.0860	2.2397	UPSP:DDX39_HUMAN	17	R.DVQEIFR.L
3265	1	2	17/18	0.2952	0.2952	2.9595	UPSP:DDX39_HUMAN	10	R.ILVATNLFGR.G
3328	1	2	16/28	0.2397	0.2397	1.9484	UPSP:MSH6_HUMAN	4	K.GNWAHSGFPEIAFGR.Y
0779	1	2	13/16	0.1062	0.1062	2.3091	UPSP:MSH6_HUMAN	4	K.IHNVGSPLK.S
0511	1	2	14/18	0.3053	0.3053	2.6456	UPSP:MSH6_HUMAN	3	R.STVDAEAVHK.L
3006	1	3	32/80	0.2958	0.2958	3.9836	UPSP:MSH6_HUMAN	3	R.TLFSTHYHSLVEDYSQNVAVR.L
3462	1	3	25/56	0.1598	0.1598	3.3560	UPSP:MSH6_HUMAN	4	R.TLVAHYPPVQVLFEK.G
4216	1	3	42/96	0.4312	0.4312	5.6567	UPSP:DNJA1_HUMAN	5	K.ITFHGEGDQEPGLEPGDIIIVLDQK.D
2488	1	2	17/24	0.4915	0.4915	3.6507	UPSP:DNJA1_HUMAN	3	K.QISQAYEVLSDAK.K
1044	1	2	17/22	0.2177	0.2177	3.1967	UPSP:DNJA1_HUMAN	3	R.ELYDKGGEQAIK.E
1880	1	2	18/24	0.3536	0.3536	3.7476	UPSP:DNJA1_HUMAN	5	R.TIVITSHPGQIVK.H
3543	1	2	18/30	0.2189	0.2189	2.6475	UPSP:NDRG1_HUMAN	3	K.SIIGMGTGAGAYILTR.F
2816	1	2	22/30	0.3515	0.3515	4.7574	UPSP:NDRG1_HUMAN	5	R.EMQDVDLAEVKPLVEK.G
1375	1	3	30/96	0.1880	0.1880	4.6537	UPSP:NDRG1_HUMAN	4	R.SHTSEGAHLDITPNSGAAGNSAGPK.S
1183	1	2	19/26	0.3812	0.3812	2.9298	UPSP:NDRG1_HUMAN	5	R.TASGSSVTSLDGTR.S
3125	1	2	16/18	0.2804	0.2804	3.5789	UPSP:RLA0_HUMAN	6	K.IIQLLDDYPK.C
4081	1	2	19/22	0.4713	0.4713	3.6615	UPSP:RLA0_HUMAN	5	K.TSFFQALGITTK.I
0719	1	2	17/20	0.3960	0.3960	3.3761	UPSP:RLA0_HUMAN	6	R.GHLENNPALEK.L
4093	1	2	22/32	0.4284	0.4284	4.2282	UPSP:RLA0_HUMAN	7	R.VLALSVETDYTFPLAEK.V
2358	1	2	19/28	0.4772	0.4772	3.8677	UPSP:RINI_HUMAN	7	K.ELSLAGNELGDEGAR.L
1998	1	2	19/28	0.3019	0.3019	4.0993	UPSP:RINI_HUMAN	6	K.ELTVSNNDINEAGVR.V
0807	1	2	11/14	0.1147	0.1147	2.0661	UPSP:RINI_HUMAN	6	R.ELALGSNK.L
2828	1	2	18/20	0.4311	0.4311	3.5451	UPSP:RINI_HUMAN	5	R.VNPALAELNLR.S
4284	1	3	31/92	0.0638	0.0638	3.3453	UPTR:Q53HU2_HUMAN	2	K.GHNGWVTQIATTPQFPDMILSASR.D
2875	1	2	19/30	0.2310	0.2310	4.0998	UPTR:Q53HU2_HUMAN	3	K.IIVDELKQEVISTSSK.A
1480	1	2	15/18	0.3193	0.3193	2.9695	UPTR:Q53HU2_HUMAN	2	R.DETNYGIPQR.A
2601	1	2	16/20	0.2813	0.2813	3.1445	UPTR:Q53HU2_HUMAN	2	R.LWDLTTGTTTR.R
3666	1	2	19/22	0.4720	0.4720	3.8975	UPTR:Q6FGB8_HUMAN	4	K.TVTAMDVVYALK.R
2897	1	2	10/14	0.1232	0.1232	2.0865	UPTR:Q6FGB8_HUMAN	5	K.VFLENVIR.D
1568	1	3	18/44	0.2517	0.2517	3.0894	UPTR:Q6FGB8_HUMAN	4	R.DNIQGITKPAIR.R
2181	1	2	17/18	0.3345	0.3345	3.5019	UPTR:Q6FGB8_HUMAN	5	R.ISGLIYEETR.G
3075	1	2	14/16	0.2125	0.2125	2.6027	UPSP:HSP72_HUMAN	10	K.LLQDFFNGK.E
1413	1	3	17/60	0.3905	0.3905	3.1151	UPSP:HSP72_HUMAN	8	K.STAGDTHLGGEDFDNR.M
3029	1	3	22/44	0.0927	0.0927	3.3017	UPSP:HSP72_HUMAN	7	R.ARFEELNADLFR.G
3303	1	2	15/18	0.3753	0.3753	2.8236	UPSP:HSP72_HUMAN	7	R.FEELNADLFR.G
1427	1	2	11/14	0.2103	0.2103	2.1504	UPSP:HNRPU_HUMAN	10	K.DIDIHEVR.I
1884	1	2	10/12	0.0754	0.0754	2.0250	UPSP:HNRPU_HUMAN	9	K.FIEIAAR.K
2889	1	2	16/22	0.2736	0.2736	3.1360	UPSP:HNRPU_HUMAN	9	K.YNILGTNTIMDK.M
2689	1	2	13/18	0.2468	0.2468	2.5857	UPSP:HNRPU_HUMAN	7	R.GYFEYIEENK.Y
2299	1	2	11/12	0.1551	0.1551	2.4166	UPSP:RS9_HUMAN	4	K.IEDFLER.R
2100	1	2	12/14	0.1602	0.1602	2.6481	UPSP:RS9_HUMAN	4	K.LIGEYGLR.N
3526	1	2	14/20	0.1127	0.1127	2.2012	UPSP:RS9_HUMAN	4	K.QVVNIPSFIVR.L
1394	1	2	11/14	0.2606	0.2606	2.3816	UPSP:RS9_HUMAN	3	R.IGVLDEGK.M
0051	1	2	12/14	0.1542	0.1542	2.0940	UPSP:MSH2_HUMAN	12	K.MSAVDGQR.Q
2986	1	2	17/20	0.3055	0.3055	3.2794	UPSP:MSH2_HUMAN	1	K.NNSFVNEIISR.I
2418	1	2	12/16	0.0881	0.0881	2.0153	UPSP:MSH2_HUMAN	10	R.QTLQEDLLR.R
0485	1	2	10/16	0.0675	0.0675	1.9821	UPSP:MSH2_HUMAN	5	R.VGAGDSQLK.G
0458	1	2	11/16	0.3212	0.3212	2.1299	UPSP:MYPT1_HUMAN	3	K.ETQTDSISR.Y
2516	1	2	16/26	0.2889	0.2889	2.6820	UPSP:MYPT1_HUMAN	5	K.TGSYGALAEITASK.E
1210	1	2	11/16	0.1053	0.1053	2.0371	UPSP:MYPT1_HUMAN	6	R.QGVDIEAAR.K
1634	1	2	14/18	0.1824	0.1824	2.1300	UPSP:MYPT1_HUMAN	3	R.SGSYSYLEER.K
4418	1	3	24/60	0.3381	0.3381	3.6291	UPSP:PMS1_HUMAN	10	K.IQDLLMSFGILKPDLR.I
2367	1	2	17/20	0.2549	0.2549	3.3926	UPSP:PMS1_HUMAN	6	K.LDELLQSQIEK.R
4423	1	2	19/30	0.4565	0.4565	4.8380	UPSP:PMS1_HUMAN	14	R.LLSSSQIITSVVSVVK.E
2388	1	2	18/24	0.3089	0.3089	3.5445	UPTR:Q9NQS8_HUMAN	5	K.DEILPTTPISEQK.G
3582	1	3	29/84	0.3842	0.3842	4.4895	UPTR:Q9NQS8_HUMAN	5	K.FVDGLMIHSGDPVNYYVDTAVR.H
2095	1	2	15/30	0.3905	0.3905	3.2087	UPTR:Q9NQS8_HUMAN	4	K.GGKPEPPAMPQPVPTA.-
2187	1	2	14/20	0.0900	0.0900	2.3764	UPSP:RL7A_HUMAN	4	K.NFGIGQDIQPK.R
2335	1	2	18/24	0.3936	0.3936	3.5249	UPSP:RL7A_HUMAN	6	R.AGVNTVTTLVENK.K
3714	1	3	31/60	0.3560	0.3560	5.0286	UPSP:RL7A_HUMAN	4	R.LKVPPAINQFTQALDR.Q
2493	1	2	18/28	0.3558	0.3558	4.0411	UPSP:HSP71_HUMAN	5	K.NQVALNPQNTVFDAK.R
3298	1	2	23/30	0.5224	0.5224	4.2478	UPSP:HSP71_HUMAN	9	R.IINEPTAAAIAYGLDR.T
2329	1	2	13/18	0.3423	0.3423	2.5083	UPSP:HSP71_HUMAN	6	R.LVNHFVEEFK.R
3497	1	3	25/76	0.2852	0.2852	3.1866	UPSP:2AAA_HUMAN	5	K.IGPILDNSTLQSEVKPILEK.L
0778	1	2	14/18	0.1426	0.1426	3.0620	UPSP:2AAA_HUMAN	6	K.LTQDQDVDVK.Y
4020	1	2	22/36	0.4522	0.4522	4.4940	UPSP:2AAA_HUMAN	6	K.SEIIPMFSNLASDEQDSVR.L
2542	1	2	17/22	0.3349	0.3349	3.0947	UPSP:2ABA_HUMAN	2	K.NAAQFLLSTNDK.T
3659	1	3	22/60	0.1605	0.1605	3.3264	UPSP:2ABA_HUMAN	1	R.VPVFRPMDLMVEASPR.R
1780	1	2	17/20	0.2552	0.2552	3.8985	UPSP:2ABA_HUMAN	2	R.VVIFQQEQENK.I
0864	1	2	11/12	0.0532	0.0532	1.8830	UPSP:MCM7_HUMAN	3	K.YGNQLVR.L
4289	1	3	31/72	0.4192	0.4192	4.3209	UPSP:MCM7_HUMAN	4	R.IAQPGDHVSVTGIFLPILR.T
3878	1	2	18/28	0.3699	0.3699	3.8113	UPSP:MCM7_HUMAN	4	R.SLEQNIQLPAALLSR.F
1964	1	2	19/20	0.3043	0.3043	3.9716	UPTR:Q59GP5_HUMAN	31	K.IGGIGTVPVGR.V
2708	1	2	19/22	0.2608	0.2608	3.2962	UPTR:Q59GP5_HUMAN	22	R.EHALLAYTLGVK.Q
2146	1	2	13/14	0.0520	0.0520	2.4228	UPTR:Q59GP5_HUMAN	29	R.LPLQDVYK.I
3258	1	3	26/64	0.2195	0.2195	3.4355	UPSP:TCPA_HUMAN	3	K.LGVQVVITDPEKLDQIR.Q
2362	1	2	15/20	0.2173	0.2173	2.3008	UPSP:TCPA_HUMAN	4	K.QAGVFEPTIVK.V
3113	1	2	19/28	0.2735	0.2735	3.5368	UPSP:TCPA_HUMAN	3	R.YINENLIVNTDELGR.D
4122	1	2	20/32	0.4878	0.4878	4.6645	UPSP:SF3B3_HUMAN	2	K.LGAVFNQVAFPLQYTPR.K
4054	1	2	16/30	0.1383	0.1383	2.5729	UPSP:SF3B3_HUMAN	3	R.FSNTGEDWYVLVGVAK.D
2943	1	2	15/22	0.1847	0.1847	2.0059	UPSP:SF3B3_HUMAN	3	R.IVPGQFLAVDPK.G
3753	1	2	16/20	0.3463	0.3463	3.2315	UPSP:TCPG_HUMAN	7	K.ELGIWEPLAVK.L
3478	1	2	18/20	0.2055	0.2055	3.3558	UPSP:TCPG_HUMAN	7	K.TAVETAVLLLR.I
3120	1	2	16/20	0.1685	0.1685	2.5595	UPSP:TCPG_HUMAN	7	R.IVLLDSSLEYK.K
1435	1	2	14/16	0.2358	0.2358	3.0447	UPTR:Q8WVX7_HUMAN	1	K.DVNQQEFVR.A
3528	1	2	14/24	0.4684	0.4684	2.5254	UPTR:Q8WVX7_HUMAN	1	K.ELAPYDENWFYTR.A
0453	1	2	15/18	0.3110	0.3110	3.3052	UPTR:Q8WVX7_HUMAN	1	R.IAGQVAAANK.K
2506	1	2	13/22	0.2780	0.2780	2.1021	UPSP:RS6_HUMAN	7	K.DIPGLTDTTVPR.R
1347	1	2	12/14	0.2453	0.2453	3.0436	UPSP:RS6_HUMAN	6	K.LIEVDDER.K
3084	1	2	20/28	0.4282	0.4282	3.5742	UPSP:RS6_HUMAN	6	R.MATEVAADALGEEWK.G
3690	1	2	17/24	0.2805	0.2805	3.2704	UPSP:DDX48_HUMAN	4	K.MLVLDEADEMLNK.G
2398	1	3	22/60	0.2686	0.2686	3.1197	UPSP:DDX48_HUMAN	13	R.GIYAYGFEKPSAIQQR.A
1699	1	2	10/14	0.0745	0.0745	2.1755	UPSP:DDX48_HUMAN	13	R.KVDWLTEK.M
2597	1	2	13/16	0.1515	0.1515	2.5723	UPTR:Q6IPX9_HUMAN	5	K.AELNEFLTR.E
2193	1	2	12/14	0.1349	0.1349	2.4619	UPTR:Q6IPX9_HUMAN	5	K.LLGGLAVR.R
2676	1	2	15/16	0.1667	0.1667	3.4230	UPTR:Q6IPX9_HUMAN	5	R.TEIIILATR.T
0812	1	2	13/18	0.1608	0.1608	2.3971	UPSP:FLNB_HUMAN	4	K.IFAQDGEGQR.I
3419	1	2	16/28	0.2034	0.2034	2.4720	UPSP:FLNB_HUMAN	4	K.SPFTVGVAAPLDLSK.I
2718	1	2	17/24	0.3454	0.3454	2.8074	UPSP:FLNB_HUMAN	2	R.IGNLQTDLSDGLR.L
0670	1	2	10/14	0.1537	0.1537	1.8102	UPTR:Q6PJ43_HUMAN	26	K.IIAPPERK.Y
2499	1	2	10/14	0.2437	0.2437	2.1601	UPTR:Q6PJ43_HUMAN	21	R.DLTDYLM*K.I
1552	1	2	15/18	0.4334	0.4334	2.7421	UPTR:Q6PJ43_HUMAN	11	R.GYSFTTTAER.E
3481	1	3	46/116	0.4827	0.4827	5.5734	UPTR:Q562X8_HUMAN	28	R.TTGIVM*DSGDGVTHTVPIYEGYALPHAILR.L
3671	1	3	38/116	0.3580	0.3580	6.0833	UPTR:Q562X8_HUMAN	28	R.TTGIVMDSGDGVTHTVPIYEGYALPHAILR.L
4360	1	2	22/32	0.4626	0.4626	4.9079	UPSP:HNRH1_HUMAN	7	R.ATENDIYNFFSPLNPVR.V
3627	1	2	21/32	0.5657	0.5657	4.2866	UPSP:HNRH1_HUMAN	3	R.STGEAFVQFASQEIAEK.A
3541	1	3	24/76	0.1948	0.1948	3.4792	UPSP:TCPE_HUMAN	2	K.ISDSVLVDIKDTEPLIQTAK.T
4432	1	2	22/30	0.4970	0.4970	5.4541	UPSP:TCPE_HUMAN	3	K.LGFAGLVQEISFGTTK.D
4148	1	2	21/24	0.4641	0.4641	5.3590	UPTR:Q9BRB1_HUMAN	7	K.MFVLDEADEMLSR.G
1479	1	3	24/52	0.3442	0.3442	3.4966	UPTR:Q9BRB1_HUMAN	2	R.KGVAINMVTEEDKR.T
3965	1	3	29/56	0.4105	0.4105	4.3901	UPSP:TBA6_HUMAN	8	R.AVFVDLEPTVIDEVR.T
4512	1	3	27/52	0.3698	0.3698	4.3422	UPSP:TBA6_HUMAN	8	R.LISQIVSSITASLR.F
2399	1	3	25/72	0.4296	0.4296	4.3386	UPTR:Q53S41_HUMAN	4	K.QTIDNSQGAYQEAFDISKK.E
1628	1	2	18/22	0.2497	0.2497	3.0591	UPTR:Q53S41_HUMAN	3	K.YLIANATNPESK.V
4320	1	3	27/84	0.1957	0.1957	3.9234	UPSP:RS17_HUMAN	2	K.LLDFGSLSNLQVTQPTVGMNFK.T
0494	1	2	15/18	0.1942	0.1942	3.4026	UPSP:RS17_HUMAN	2	R.LGNDFHTNKR.V
2642	1	2	16/28	0.2570	0.2570	3.3559	UPSP:TBB4Q_HUMAN	9	R.AVLVDLEPGTM*DSVR.S
3161	1	2	21/28	0.3826	0.3826	3.9082	UPSP:TBB4Q_HUMAN	9	R.AVLVDLEPGTMDSVR.S
4190	1	2	23/38	0.5310	0.5310	4.6632	UPTR:Q53G25_HUMAN	2	K.WSTDDVQINDISLQDYIAVK.E
1569	1	2	13/16	0.1059	0.1059	2.4544	UPTR:Q53G25_HUMAN	2	R.QAVDVSPLR.R
2036	1	2	14/16	0.3962	0.3962	3.4626	GP:BC011752_1	Â	K.DFTEDTIPR.T
1489	1	2	16/18	0.3436	0.3436	3.5488	GP:BC011752_1	Â	R.KDFTEDTIPR.T
3895	1	2	17/30	0.1118	0.1118	3.5061	UPSP:U2AF2_HUMAN	1	K.LFIGGLPNYLNDDQVK.E
4154	1	3	25/84	0.2519	0.2519	3.4893	UPSP:U2AF2_HUMAN	1	R.SVDETTQAMAFDGIIFQGQSLK.I
1379	1	3	26/48	0.4613	0.4613	3.2939	UPTR:Q9BYX7_HUMAN	15	K.QEYDESGPSIVHR.K
3281	1	2	19/30	0.4007	0.4007	3.6966	UPTR:Q9BYX7_HUMAN	25	K.SYELPDGQVITIGNER.F
3924	1	2	17/28	0.3818	0.3818	3.6130	UPSP:RS24_HUMAN	2	K.TTGFGMIYDSLDYAK.K
4425	1	2	17/22	0.3918	0.3918	3.3109	UPSP:RS24_HUMAN	2	K.TTPDVIFVFGFR.T
3337	1	2	18/20	0.4637	0.4637	3.7615	UPSP:RS23_HUMAN	1	K.VANVSLLALYK.G
0962	1	3	21/44	0.1836	0.1836	3.1376	UPSP:RS23_HUMAN	1	R.KGHAVGDIPGVR.F
3551	1	2	18/24	0.3273	0.3273	4.1472	UPSP:RL18_HUMAN	3	K.ILTFDQLALDSPK.G
2129	1	2	16/20	0.2772	0.2772	2.7514	UPSP:RL18_HUMAN	3	R.TNSTFNQVVLK.R
0827	1	2	18/20	0.3359	0.3359	3.4215	GP:AY891365_1	Â	K.TPGPGAQSALR.A
1798	1	2	17/24	0.3837	0.3837	3.4144	GP:AY891365_1	Â	R.IEDVTPIPSDSTR.R
2326	1	2	16/18	0.3702	0.3702	2.9073	GP:AY892659_1	Â	K.DVNAAIAAIK.T
4188	1	2	20/28	0.1690	0.1690	3.5644	GP:AY892659_1	Â	R.AVFVDLEPTVIDEIR.N
0386	1	2	17/24	0.2148	0.2148	2.8771	UPSP:COPA_HUMAN	3	K.DADSQNPDAPEGK.R
3095	1	2	17/24	0.1492	0.1492	3.3461	UPSP:COPA_HUMAN	2	R.DADSITLFDVQQK.R
1599	1	2	19/26	0.2923	0.2923	3.9787	UPSP:RL5_HUMAN	7	K.GAVDGGLSIPHSTK.R
0923	1	2	11/18	0.1083	0.1083	2.2000	UPSP:RL5_HUMAN	7	R.RFPGYDSESK.E
1061	1	2	16/20	0.4087	0.4087	2.7559	UPSP:PCBP2_HUMAN	5	K.IANPVEGSTDR.Q
3463	1	2	16/24	0.3552	0.3552	3.3865	UPSP:PCBP2_HUMAN	5	R.IITLAGPTNAIFK.A
3087	1	2	15/20	0.1806	0.1806	2.9364	UPSP:K6PL_HUMAN	11	K.GQLESIVENIR.I
2572	1	3	27/68	0.1408	0.1408	3.1458	UPSP:K6PL_HUMAN	8	R.TNVLGHLQQGGAPTPFDR.N
4288	1	2	16/30	0.2871	0.2871	3.3144	UPSP:SAHH2_HUMAN	10	R.EIEIAEQDMSALISLR.K
2156	1	2	13/14	0.1720	0.1720	2.7668	UPSP:SAHH2_HUMAN	18	R.TPELTWER.V
3759	1	2	15/20	0.2661	0.2661	3.0419	UPTR:Q9NXD9_HUMAN	2	K.ILLTQENPFFR.K
3793	1	2	18/34	0.0706	0.0706	2.9806	UPTR:Q9NXD9_HUMAN	1	K.SEGALELADVSNELPGLK.W
4491	1	2	17/24	0.4053	0.4053	3.4005	UPSP:RS2_HUMAN	7	K.SLEEIYLFSLPIK.E
2673	1	2	14/22	0.1551	0.1551	2.5643	UPSP:RS2_HUMAN	7	K.SPYQEFTDHLVK.T
3055	1	2	15/16	0.2040	0.2040	2.9391	UPTR:Q5GGW2_HUMAN	Â	K.IAFAITAIK.-
1229	1	2	14/16	0.1994	0.1994	2.8753	UPTR:Q5GGW2_HUMAN	Â	R.VLNTNIDGR.R
1872	1	2	12/14	0.0656	0.0656	1.8561	UPSP:RL7_HUMAN	7	K.SVNELIYK.R
3892	1	2	17/26	0.4048	0.4048	3.8792	UPSP:RL7_HUMAN	7	R.IVEPYIAWGYPNLK.S
1276	1	2	16/20	0.2154	0.2154	3.5042	UPTR:Q498Y6_HUMAN	1	K.EAIEGTYIDKK.C
2149	1	2	10/12	0.1439	0.1439	2.2169	UPTR:Q498Y6_HUMAN	2	R.DYLHYIR.K
0935	1	2	12/14	0.1891	0.1891	2.7879	UPSP:RL26L_HUMAN	3	K.DDEVQVVR.G
0672	1	2	12/16	0.2442	0.2442	2.6891	UPSP:RL26L_HUMAN	3	R.KDDEVQVVR.G
1161	1	2	13/20	0.1293	0.1293	2.2196	UPSP:RS4Y1_HUMAN	9	K.GIPHLVTHDAR.T
1327	1	3	25/48	0.3175	0.3175	3.2374	UPSP:RS4Y1_HUMAN	6	R.ERHPGSFDVVHVK.D
2135	1	2	13/20	0.2944	0.2944	2.6580	UPTR:Q9BS10_HUMAN	2	-.LTPEEEEILNK.K
1328	1	2	14/16	0.2098	0.2098	2.6869	UPTR:Q9BS10_HUMAN	2	R.ADGYVLEGK.E
2724	1	2	17/20	0.4649	0.4649	2.8288	UPTR:Q6IPX4_HUMAN	1	K.GPLQSVQVFGR.K
3917	1	2	12/18	0.2731	0.2731	2.2408	UPTR:Q6IPX4_HUMAN	1	K.LLEPVLLLGK.E
2073	1	2	13/18	0.1764	0.1764	2.1444	GP:AY892156_1	Â	K.LITPAVVSER.L
2615	1	2	15/16	0.2863	0.2863	2.8762	GP:AY892156_1	Â	R.AALQELLSK.G
2366	1	2	12/12	0.1144	0.1144	2.0664	UPSP:NOLA1_HUMAN	1	K.FYIDPYK.L
2697	1	2	14/16	0.3629	0.3629	2.9272	UPSP:NOLA1_HUMAN	1	K.VDEIFGQLR.D
2396	1	2	14/16	0.0970	0.0970	2.4362	UPSP:H2A1A_HUMAN	33	R.AGLQFPVGR.I
1524	1	2	12/12	0.1111	0.1111	2.5047	UPSP:H2A1A_HUMAN	30	R.HLQLAIR.N
1470	1	2	14/20	0.0959	0.0959	2.6207	UPTR:Q6ZSQ4_HUMAN	27	K.EITALAPSTM*K.I
2019	1	2	16/20	0.1597	0.1597	2.2815	UPTR:Q6ZSQ4_HUMAN	27	K.EITALAPSTMK.I
3686	1	2	14/20	0.2051	0.2051	2.5759	UPSP:PLEC1_HUMAN	10	R.IISLETYNLLR.E
0816	1	2	13/18	0.1364	0.1364	2.2136	UPSP:PLEC1_HUMAN	10	R.LLEAAAQSTK.G
1940	1	2	13/14	0.3074	0.3074	2.4744	UPSP:RS15A_HUMAN	1	K.IVVNLTGR.L
2313	1	2	14/16	0.1668	0.1668	2.2718	UPSP:RS15A_HUMAN	1	K.WQNNLLPSR.Q
3059	1	2	13/16	0.2408	0.2408	2.8111	UPSP:NOL1_HUMAN	4	R.IQDIVGILR.D
3344	1	2	11/28	0.1895	0.1895	1.9248	UPSP:NOL1_HUMAN	4	R.LVPTGLDFGQEGFTR.F
0769	1	2	13/18	0.1389	0.1389	2.6004	UPSP:RL23_HUMAN	2	K.GSAITGPVAK.E
2675	1	2	12/14	0.1730	0.1730	2.1012	UPSP:RL23_HUMAN	3	K.NLYIISVK.G
3204	1	2	17/30	0.1478	0.1478	2.4560	UPSP:TOP1_HUMAN	3	K.GPVFAPPYEPLPENVK.F
2749	1	2	15/22	0.2446	0.2446	2.2322	UPSP:TOP1_HUMAN	4	K.HLQDLMEGLTAK.V
2150	1	2	13/18	0.1833	0.1833	2.6500	UPSP:NUCL_HUMAN	5	K.EVFEDAAEIR.L
0272	1	2	13/14	0.1129	0.1129	2.0189	UPSP:NUCL_HUMAN	6	K.QGTEIDGR.S
2768	1	2	13/18	0.1396	0.1396	2.5751	UPSP:RL27A_HUMAN	4	K.LWTLVSEQTR.V
2502	1	2	13/20	0.1029	0.1029	2.0622	UPSP:RL27A_HUMAN	4	K.TGAAPIIDVVR.S
2420	1	2	12/14	0.2707	0.2707	2.2586	UPTR:Q6NXR8_HUMAN	2	K.APAMFNIR.N
1697	1	2	12/14	0.2030	0.2030	2.2245	UPTR:Q6NXR8_HUMAN	2	K.TTDGYLLR.L
0551	1	2	14/20	0.3799	0.3799	2.2778	UPSP:H2B1A_HUMAN	38	K.QVHPDTGISSK.A
0605	1	2	10/12	0.0524	0.0524	1.8878	UPSP:H2B1A_HUMAN	37	R.EIQTAVR.L
3308	1	3	33/56	0.4386	0.4386	5.4468	UPSP:DDX20_HUMAN	6	R.IPFNQALVFSNLHSR.A
1149	1	2	21/26	0.4186	0.4186	5.3600	UPTR:Q53RR5_HUMAN	2	K.AVTEQGAELSNEER.N
4343	1	2	20/32	0.4340	0.4340	4.8545	UPSP:XPO2_HUMAN	1	K.LLQTDDEEEAGLLELLK.S
4481	1	2	22/28	0.5319	0.5319	4.8459	UPSP:TCPB_HUMAN	4	R.LTSFIGAIAIGDLVK.S
3576	1	2	24/30	0.4391	0.4391	4.5114	UPSP:TCPD_HUMAN	3	R.ALIAGGGAPEIELALR.L
3269	1	3	29/84	0.3249	0.3249	4.4647	UPSP:DKC1_HUMAN	4	R.TGFINLDKPSNPSSHEVVAWIR.R
3028	1	2	20/24	0.4761	0.4761	4.3886	UPSP:RBBP7_HUMAN	6	K.TPSSDVLVFDYTK.H
2579	1	2	21/34	0.3485	0.3485	4.3323	UPTR:Q562P9_HUMAN	26	R.VAPEEHPVLLTEAPLNPK.A
4008	1	3	31/56	0.3518	0.3518	4.3196	UPTR:Q5JP53_HUMAN	5	R.ALTVPELTQQVFDAK.N
0817	1	2	19/20	0.1258	0.1258	4.2600	UPTR:Q4R5P9_MACFA	Â	K.AAAAAAALEAK.S
3060	1	3	30/76	0.2850	0.2850	4.2355	UPSP:IMDH2_HUMAN	1	K.DKYPNLQVIGGNVVTAAQAK.N
2482	1	2	18/28	0.4973	0.4973	4.1520	UPTR:Q9NXF7_HUMAN	Â	R.TEPINTTYSYTDFQK.A
3855	1	2	20/28	0.4494	0.4494	4.1373	UPSP:H2B1B_HUMAN	33	K.AM*GIM*NSFVNDIFER.I
2470	1	2	18/24	0.3244	0.3244	4.1144	UPTR:Q7Z5A3_HUMAN	1	K.NREPVQLETLSIR.G
2373	1	2	18/24	0.4313	0.4313	4.0772	UPSP:DHX15_HUMAN	1	R.EVDDLGPEVGDIK.I
2695	1	2	18/26	0.4506	0.4506	3.9814	UPTR:Q5VVC8_HUMAN	8	K.VLEQLTGQTPVFSK.A
3357	1	2	20/24	0.3135	0.3135	3.9278	UPSP:RL10_HUMAN	9	K.FNADEFEDMVAEK.R
3011	1	2	15/26	0.3771	0.3771	3.8448	UPSP:SART3_HUMAN	1	R.TEGSLEDWDIAVQK.T
3140	1	3	27/48	0.2928	0.2928	3.8263	UPSP:RL38_HUMAN	1	R.KIEEIKDFLLTAR.R
4155	1	3	32/88	0.3190	0.3190	3.7988	UPTR:Q2NL65_HUMAN	3	R.LGPLNLQDPFDLSHNVAANVTSR.V
2975	1	2	17/24	0.3040	0.3040	3.7970	UPTR:Q1JQ76_HUMAN	4	K.KYDAFLASESLIK.Q
4261	1	2	17/30	0.4172	0.4172	3.7420	UPTR:Q6DHX9_HUMAN	1	R.GTTVSVKQLFSTLPVR.H
4302	1	2	19/26	0.4114	0.4114	3.7417	UPSP:MCM3_HUMAN	3	R.LLNNAFEELVAFQR.A
3379	1	3	28/84	0.2053	0.2053	3.7345	UPTR:Q9UHX1_HUMAN	5	K.VGRPSNIGQAQPIIDQLAEEAR.A
1368	1	2	20/26	0.4466	0.4466	3.7209	GP:AY891859_1	Â	K.GTAAAAAAAAAAAK.V
4369	1	2	18/30	0.3461	0.3461	3.7149	UPTR:Q6ICQ4_HUMAN	2	K.AAGVNVEPFWPGLFAK.A
1191	1	2	19/20	0.2820	0.2820	3.7122	UPSP:HSP76_HUMAN	3	R.VEILANDQGNR.T
1043	1	2	20/28	0.3741	0.3741	3.6300	UPSP:RL29_HUMAN	4	K.AQAAAPASVPAQAPK.R
1335	1	3	26/48	0.2360	0.2360	3.5812	UPSP:RIOK1_HUMAN	6	R.SSQDHVDEEVFKR.A
2324	1	2	18/24	0.4469	0.4469	3.4957	UPSP:HS70L_HUMAN	27	R.TTPSYVAFTDTER.L
2430	1	2	20/24	0.2492	0.2492	3.4640	GP:AE006463_8	3	R.LAPDYDALDVANK.I
3096	1	2	13/24	0.4274	0.4274	3.4561	UPSP:AKP8L_HUMAN	2	K.QTADFLQEYVTNK.T
4439	1	3	29/84	0.3998	0.3998	3.4439	UPSP:AHSA1_HUMAN	2	R.VFTTQELVQAFTHAPATLEADR.G
2343	1	2	16/18	0.2342	0.2342	3.4314	UPSP:SMD3_HUMAN	Â	R.VAQLEQVYIR.G
4082	1	2	21/26	0.3857	0.3857	3.4205	UPSP:K1967_HUMAN	3	K.VLLLSSPGLEELYR.C
1465	1	3	24/40	0.2751	0.2751	3.4135	GP:AY892286_1	Â	K.TPVEPEVAIHR.I
2859	1	3	28/68	0.2821	0.2821	3.4076	UPSP:FXR1_HUMAN	2	R.KVPGVTAIELDEDTGTFR.I
2192	1	3	21/56	0.4016	0.4016	3.3779	UPSP:RL21_HUMAN	4	R.VYNVTQHAVGIVVNK.Q
4350	1	3	28/84	0.2565	0.2565	3.3624	UPSP:RL3_HUMAN	9	K.SINPLGGFVHYGEVTNDFVMLK.G
4403	1	2	14/16	0.1041	0.1041	3.3550	UPTR:Q5TZB9_HUMAN	4	R.IAIYELLFK.E
2946	1	2	14/16	0.2704	0.2704	3.2976	UPTR:Q53HV1_HUMAN	3	R.LSNIFVIGK.G
2984	1	2	17/22	0.3220	0.3220	3.2888	UPSP:KCTD5_HUMAN	Â	R.LNVGGTYFLTTR.Q
3645	1	2	17/30	0.4039	0.4039	3.2853	UPSP:FLNA_HUMAN	11	R.VTYTPMAPGSYLISIK.Y
3207	1	2	15/20	0.1908	0.1908	3.2066	UPSP:DDX17_HUMAN	20	R.MLDMGFEPQIR.K
1926	1	2	13/22	0.2652	0.2652	3.1805	UPSP:PRS4_HUMAN	5	K.AVANQTSATFLR.V

[illegible]	1	2	[illegible]	[illegible]	[illegible]	[illegible]	[illegible]	[illegible]	[illegible]	
1485	1	2	15/18	0.3096	0.3096	3.1608	UPSP:RL35_HUMAN	4	R.VLTVINQTQK.E	
3739	1	2	17/24	0.3865	0.3865	3.1542	UPSP:LRP4_HUMAN	3	K.ALGPEPVLLFANR.I	
0650	1	2	15/18	0.2175	0.2175	3.1238	UPTR:Q6DRA9_HUMAN	2	R.DAVTYTEHAK.R	
0499	1	3	21/40	0.1743	0.1743	3.1111	UPSP:PAIRB_HUMAN	9	R.RPDQQLQGEGK.I	
4066	1	2	17/28	0.3456	0.3456	3.1033	UPSP:IMB1_HUMAN	1	K.LAATNALLNSLEFTK.A	
3285	1	2	14/28	0.2155	0.2155	3.0835	UPSP:DDX21_HUMAN	6	K.EGAFSNFPISEETIK.L	
2743	1	2	13/14	0.2771	0.2771	3.0762	UPTR:Q5T8W0_HUMAN	1	K.VFQFLNAK.C	
2730	1	2	18/22	0.2900	0.2900	3.0366	UPTR:Q9H853_HUMAN	Â	R.QIFHPEQLITGK.E	
1418	1	2	14/16	0.2588	0.2588	3.0342	UPTR:Q6IRZ0_HUMAN	Â	R.SAINEVVTR.E	
4203	1	2	14/28	0.3130	0.3130	2.9484	UPSP:PRS10_HUMAN	1	R.EVIELPLTNPELFQR.V	
3425	1	2	13/14	0.2129	0.2129	2.9403	UPTR:Q4VXZ3_HUMAN	1	K.IPDWFLNR.Q	
2338	1	2	13/16	0.3246	0.3246	2.9101	UPTR:Q5VVD0_HUMAN	5	K.YDGIILPGK.-	
1082	1	2	18/20	0.4011	0.4011	2.8389	UPTR:Q567Q7_HUMAN	4	R.AVVGVVAGGGR.I	
1976	1	2	14/20	0.1391	0.1391	2.8155	GP:AY893966_1	Â	K.LSHETVTIELK.N	
3436	1	2	19/30	0.2600	0.2600	2.8052	UPTR:Q6S376_HUMAN	8	R.APVPASELLASGVLSR.A	
3675	1	2	16/24	0.1977	0.1977	2.7848	UPSP:MCM5_HUMAN	5	R.LAALPNVYEVISK.S	
0930	1	2	14/18	0.1270	0.1270	2.7670	UPSP:LMNA_HUMAN	10	R.ITESEEVVSR.E	
4498	1	2	11/34	0.2528	0.2528	2.7657	UPSP:EF2_HUMAN	2	R.ALLELQLEPEELYQTFQR.I	
3915	1	2	13/26	0.1987	0.1987	2.7511	UPTR:Q8TBW2_HUMAN	4	K.LLTLLNSYSPPELR.N	
0578	1	2	13/20	0.1305	0.1305	2.7453	UPSP:CAYP2_HUMAN	Â	K.LDAEIVAAEKK.K	
2570	1	2	14/24	0.2980	0.2980	2.6158	UPSP:RL13_HUMAN	5	K.LATQLTGPVMPVR.N	
3294	1	2	15/22	0.2731	0.2731	2.5873	UPSP:RLA2_HUMAN	2	K.NIEDVIAQGIGK.L	
4037	1	2	14/28	0.2422	0.2422	2.5669	UPTR:Q5JVH5_HUMAN	4	R.DISEASVFDAYVLPK.L	
3687	1	2	14/20	0.2918	0.2918	2.5572	UPSP:G6PD_HUMAN	11	R.GGYFDEFGIIR.D	
2479	1	2	12/16	0.3119	0.3119	2.5519	UPTR:Q8WTY3_HUMAN	1	R.YSVDIPLDK.T	
1563	1	2	13/14	0.2315	0.2315	2.1697	UPSP:KPYM_HUMAN	13	R.APIIAVTR.N	

17567.2.sumlist Save data
